# Analysis of the interrelationship between precipitation and confirmed dengue cases in the city of Recife (Brazil) covering climate and public health information

**DOI:** 10.3389/fpubh.2024.1456043

**Published:** 2024-10-23

**Authors:** Iuri Valerio Graciano Borges, Anwar Musah, Livia Marcia Mosso Dutra, Merve Tunali, Clarisse Lins Lima, Mehmet Meric Tunali, Ana Clara Gomes da Silva, Aisha Aldosery, Giselle Machado Magalhães Moreno, Wellington P. dos Santos, Tiago Massoni, Orhan Yenigün, Patty Kostkova, Rosmeri Porfirio da Rocha, Luiza C. Campos, Tercio Ambrizzi

**Affiliations:** ^1^Department of Atmospheric Sciences, Institute of Astronomy, Geophysics and Atmospheric Sciences (IAG), University of São Paulo, São Paulo, Brazil; ^2^Department of Civil Environmental and Geomatic Engineering, University College London, London, United Kingdom; ^3^Geospatial Analytics and Computing Group (GSAC), Department of Geography, University College London, London, United Kingdom; ^4^Institute of Environmental Sciences, Bogazici University, Istanbul, Türkiye; ^5^Polytechnic School of Pernambuco, University of Pernambuco (Poli-UPE), Recife, Pernambuco, Brazil; ^6^Department of Biomedical Engineering, Federal University of Pernambuco, Recife, Brazil; ^7^Centre for Digital Public Health and Emergencies, Institute for Risk and Disaster Reduction, University College London, London, United Kingdom; ^8^Department Systems and Computing, Federal University of Campina Grande, Campina Grande, Paraiba, Brazil; ^9^School of Engineering, European University of Lefke, Lefke, North Cyprus, Türkiye; ^10^Centre for Urban Sustainability and Resilience, University College London, London, United Kingdom

**Keywords:** *Aedes aegypti*, arboviruses, rainfall, regression model, quantile analysis, serotypes, dengue

## Abstract

Large-scale epidemics of arboviruses, such as dengue, have heightened societal awareness regarding the necessity of combating the primary transmission vectors. Equally critical is the identification of environmental conditions and variables that influence vector population dynamics. *Aedes aegypti*, the primary vector of arboviruses such as dengue and Zika in Brazil, is closely associated with the climatic and geographical conditions of urban environments. This study examines the relationship between precipitation and confirmed dengue cases in Recife (Brazil), employing regression and quantile analyses to evaluate the influence of meteorological conditions on the disease’s spread. The findings reveal a direct correlation between monthly averages of precipitation and confirmed cases, although this is apparent only when excluding years of epidemic peaks. The highest number of cases generally aligns with the rainy season, and the lowest with the dry season, with weak, moderate and strong precipitation events being closely linked to increased dengue incidence. However, notable discrepancies were identified: four out of six major outbreaks occurred in drier months, challenging the assumption of a straightforward relationship between rainfall and dengue incidence. These findings underscore the multifaceted nature of dengue dynamics, suggesting that while precipitation plays a significant role, other factors, including serotype circulation and broader climatic phenomena, are equally critical in driving outbreaks. This complexity highlights the need for a more comprehensive understanding of the mechanisms influencing dengue epidemics.

## 1 Introduction

Large-scale epidemics of arboviruses, such as dengue, have raised an alert for society regarding the fight against their main transmission vectors (1–3). Additionally, there is a need to identify the conducive environments, conditions and variables that have greater influence and effect on these vector populations.

*Aedes aegypti* is recognized as one of the primary and most effective vectors of arboviruses (3, 45), responsible for significant epidemics in Brazil, including Zika, dengue, and chikungunya. Despite its widespread adaptation to human-influenced environments, particularly in large urban centres, studies have demonstrated that *A. aegypti* populations from distinct geographic regions exhibit varying levels of vectorial competence. These differences occur even among populations residing near humans, underscoring the influence of local environmental and genetic factors on the mosquito’s ability to transmit pathogens (2, 4–6). For this reason, combating the proliferation of populations of larvae of this insect is extremely difficult. It utilises stagnant water sources for the generation of breeding sites, which are present in both private and public buildings. This requires substantial government investment in control and information campaigns about the mosquito, as well as the cooperation of the entire community of residents (2, 4–6, 42).

Dengue in Brazil has become an epidemiological problem since the 1990s, marked by an increase in the number of cases and its presence on a national scale in the following years (2, 4, 5). The disease is caused by a virus, and it exhibits four different immune responses in the infected individual, known as serotypes (7, 8). Their introduction into a population without previous exposure has the potential to trigger explosive epidemics. The interrelationship between these serotypes in the same population is still not fully understood, but studies indicate that their co-circulation has the potential to cause more severe forms of the disease and even epidemics (4, 5, 8, 9).

Climate factors also influence the presence and abundance of mosquito populations (5). In the city of Recife (Pernambuco state), located on the coast of the northeast region of Brazil, precipitation is demonstrated to be the most predominant meteorological variable in studies that correlate it with the increase and decline of *A. aegypti* mosquito larvae populations (10).

Investigations seeking to identify the points of convergence between meteorological and health factors are still sparse. However, the work of Lima et al. (11), for example, shows a good correlation of precipitation and oceanic conditions with dengue cases, aiming to predict this variable through multiple linear regression. Silva et al. (12) also show good results in predicting cases of this arbovirus in the city of Recife, also applying the multiple linear regression method combined with machine learning on meteorological data, including precipitation, temperature, and humidity.

The present study focuses on understanding the pattern and limits of meteorological variables concerning dengue case counts in the city of Recife. For this purpose, the monthly and seasonal patterns for precipitation and case counts were described, aiming to identify possible confluences in both time series. Furthermore, to determine which type of precipitation has more influence, a quantile analysis was conducted. Simulations using a negative binomial regression model were also performed for the same objective. To the best of our knowledge, this is the first time that the negative binomial regression model is used for predicting dengue case counts, incorporating not only meteorological variables but also serotype information as explanatory variables.

## 2 Methods

### 2.1 Study area

**Figure 1:**
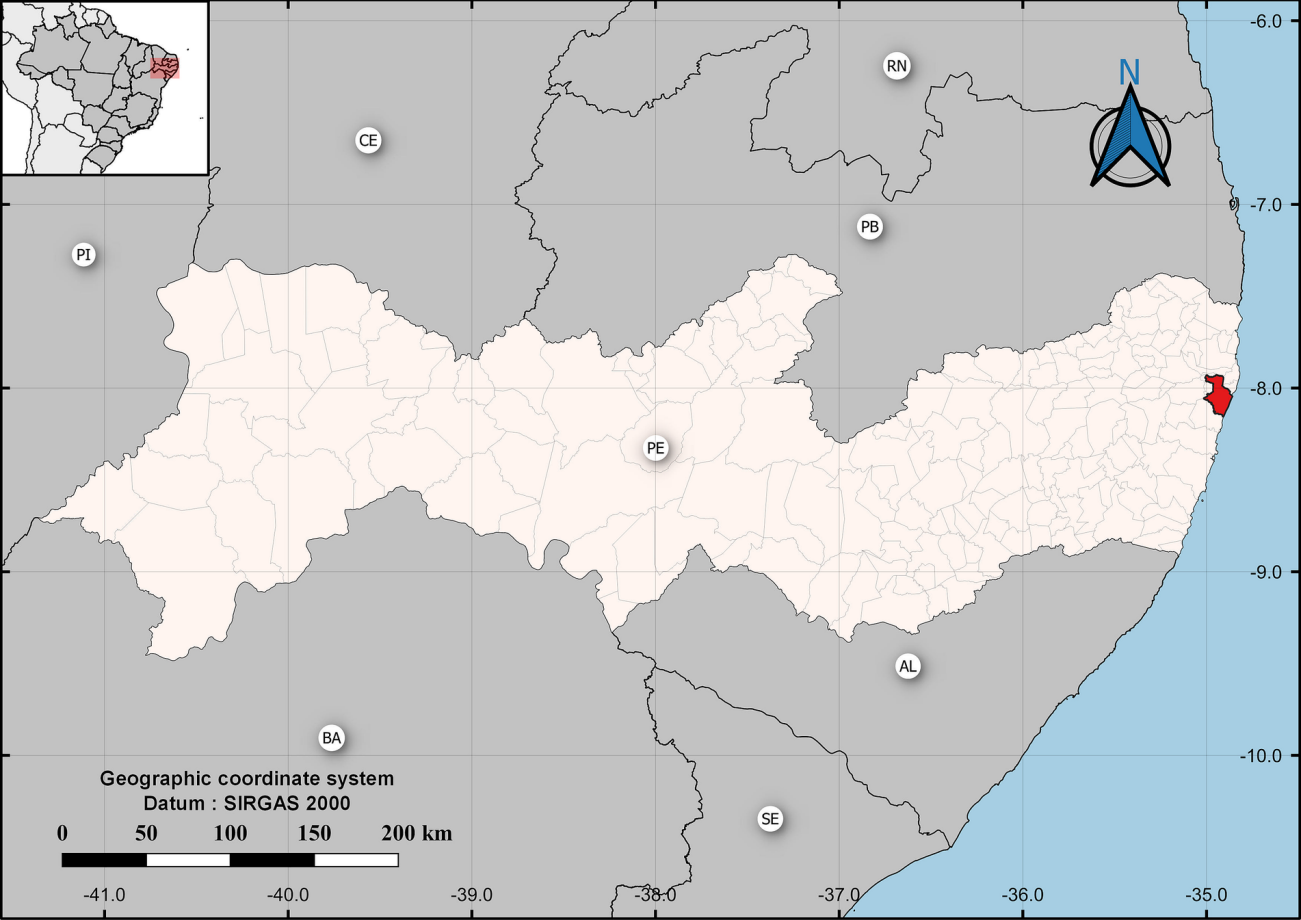
Geographic location of the study region, with the city of Recife highlighted (red).

The municipality of Recife is the capital city of the state of Pernambuco, localized in the northeast region of Brazil, as highlighted in Figure 1.

The climatology of events on the synoptic scale for the region is very diverse. Reboita et al. (13) carried out a literature review on precipitation regimes in South America and described the events that influence the annual rainfall regime in the city. The maximum rainfall in this location occurs in the first half of the year, as was also highlighted by Alvarez et al. (46).

In our study area, it is described that the South Atlantic Subtropical High (SASH) has a constant role on the transport of moisture to this location, and the intensity may vary according to the positioning of this system over the Atlantic (14). The eastern wave disturbances (EWD) also play a key role in bringing precipitation to the region, being defined as disturbances generated in the pressure field with a break in the cloud cover in the tropical region of the globe, with a shift from east to west (15–17). The High-Level Cyclonic Vortices (HLCVs) are also important for the influence on the rainfall regime, being defined as a region of high level vortex driven by the increase of warm advection in 850 hPa in the southeastern coast of Brazil linked to the displacement of a cold front heading towards the tropics (18).

**Figure 2:**
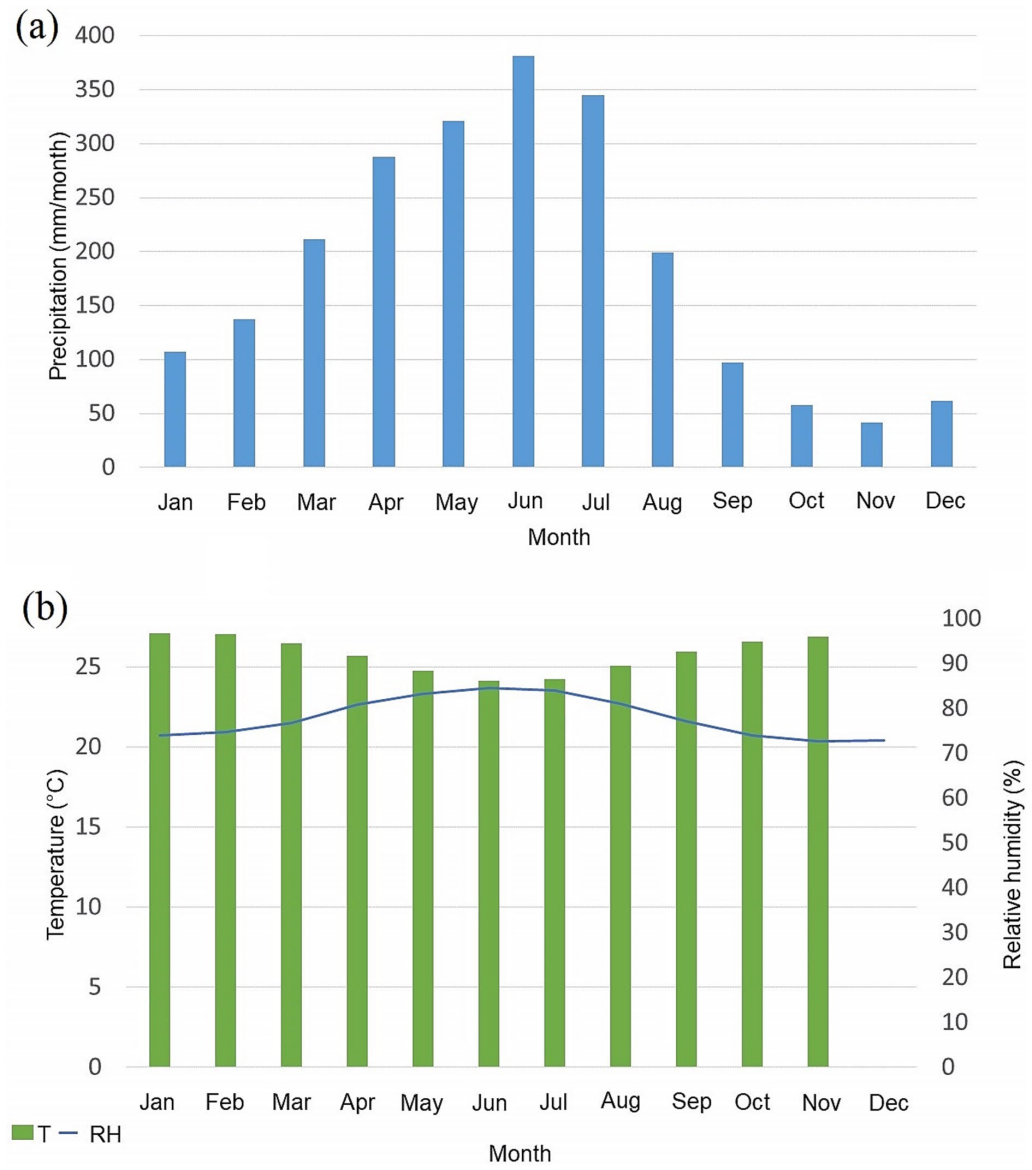
Monthly averages for Recife from the INMET conventional station (Curado) with the data covering from 1980 to 2018 referring to the variables. (a) Precipitation, (b) temperature (bars) and relative humidity (blue line).

The city of Recife has its rainy season from May to July, while December, January and February have the lowest rainfall. Due to its geographic position (i.e., on the coast and close to the Equator), the temperature and relative humidity of the air do not change significantly throughout the year, remaining practically constant (Figure 2) (46).

### 2.2 Data

For the study area, the numbers of notifications of confirmed dengue cases were obtained in the period from 2001 to 2019 through the public platform provided by the Brazilian Unified Health System (SUS in Portuguese), DATASUS, which receives the information and counts registered in the health units from the Notification Disease Information System (SINAN in Portuguese). These numbers are available on a monthly temporal frequency, representing the notifications per health unit referring to the patient’s municipality of residence (i.e., Recife). For considering only confirmed cases, it was necessary to discard all classifications that did not confirm the dengue occurrence. Although for the period of study (i.e., from 2001 to 2019) there are two kinds of forms to register the occurrence, one regarding 2001–2013 and another from 2014 onwards, the “discarded,” “ignored/white” and/or “inconclusive” were not included in the counts for this study, being considered all other classifications. Thus, for the first form covering 2001–2013, its considered an occurrence the classifications classic dengue, dengue with complications, dengue haemorrhagic fever (DHF) and dengue shock syndrome (DSS). From the second form, covering 2014 onwards, it is counted dengue, dengue with warning signs, severe dengue.

Data from the DATASUS public platform, covering the period from 2013 to 2019, were used to analyse the isolated serotype count per patient. For the period from 2001 to 2012, which is not covered by DATASUS, data from case studies analysing the epidemiological situation in Recife were used. The information was sourced from Montenegro et al. (19) for 2002, Castanha (20) for 2005 and 2006, and Silva et al. (8) for 2007 to 2009. In years where data were unavailable, the DENV-1 serotype was assumed to be predominant in non-epidemic periods, as discussed by Barreto and Teixeira (4). To account for the impact of each serotype on case counts and to simulate the interrelation and predominance of each serotype in the population, percentage values calculated from the total count were applied.

The meteorological data were collected from the conventional station of the National Institute of Meteorology (INMET in Portuguese) located in Recife (Curado – code 82900, latitude −8.05916666 and longitude −34.95916666) for the same period available with the dengue data monthly (2001–2019). The variables collected for analysis include insolation, precipitation, temperature, days of precipitation, atmospheric pressure and relative humidity.

### 2.3 Categorizing precipitation

To analyse which type of daily precipitation has the most significant impact on dengue cases in Recife, a classification of this variable was performed using the quantile technique (21–23). This calculation was employed by Monteiro et al. (21) and Souza et al. (24) to classify precipitation in Recife. Initially, a dry day is considered any value lower than 2 mm/day, as values of this magnitude do not generate a significant impact with regard to water supply and soil infiltration (24).

Afterward, the remaining values were arranged in ascending order, and Equations 1, 2 were applied.


(1) $$Q\left(P\right)=y_{i}+\left[\frac{\left(P-P_{i}\right)}{\left(P_{i+1}-P_{i}\right)}\right]*\left(y_{i+1}-y_{i}\right)$$

(2) $$P_{i}=\frac{i}{\left(N+1\right)}$$

Where *Q*(*P*) is the quantile corresponding to the quantile order *P*, *i* the order number of each data arranged in ascending order, *y* the precipitation value (mm) for each order, *N* the number of elements in the series and P_i_ the quantile order.

In the study by Souza et al. (24), an order of classification of daily precipitation is defined with the quantile technique for Recife, as illustrated in Table 1, which was also applied in the present work. For the characterization of monthly rainfall, the same method was used but for different quantile intervals (21) as presented in Table 2.

**Table 1:** Classification order of daily precipitation from 2001 to 2019 using the quantile technique for the city of Recife—PE with the appropriate classes and probabilities, where Qy (*y* = 5, 25, 50, 75, and 95%) is the quantile limit used for each classification and *y* is the precipitation value of the sample.

Classification	Quantile
Dry day	*y* < Q0.05
Very weak	Q0.05 ≤ *y* < Q0.25
Weak	Q0.25 ≤ *y* < Q0.50
Moderate	Q0.50 ≤ *y* < Q0.75
Strong	Q0.55 ≤ *y* < Q0.95
Very strong	*y* ≥ Q0.95

Adapted from Souza et al. (24).

**Table 2:** Classification order of monthly rainfall through the quantile technique for the city of Recife—PE with the appropriate classes and probabilities, with Qy (*y* = 15, 35, 65, and 85%) being the quantile limit used for each classification and *y* the sampling rainfall value.

Classification	Quantile
Very dry	*y* ≤ Q0.15
Dry	Q0.15 < *y* ≤ Q0.35
Normal	Q0.35 < *y* < Q0.65
Rainy	Q0.65 ≤ *y* < Q0.85
Very rainy	*y* ≥ Q0.85

Adapted from Monteiro et al. (21).

### 2.4 Negative Binomial regression models

**Figure 3:**
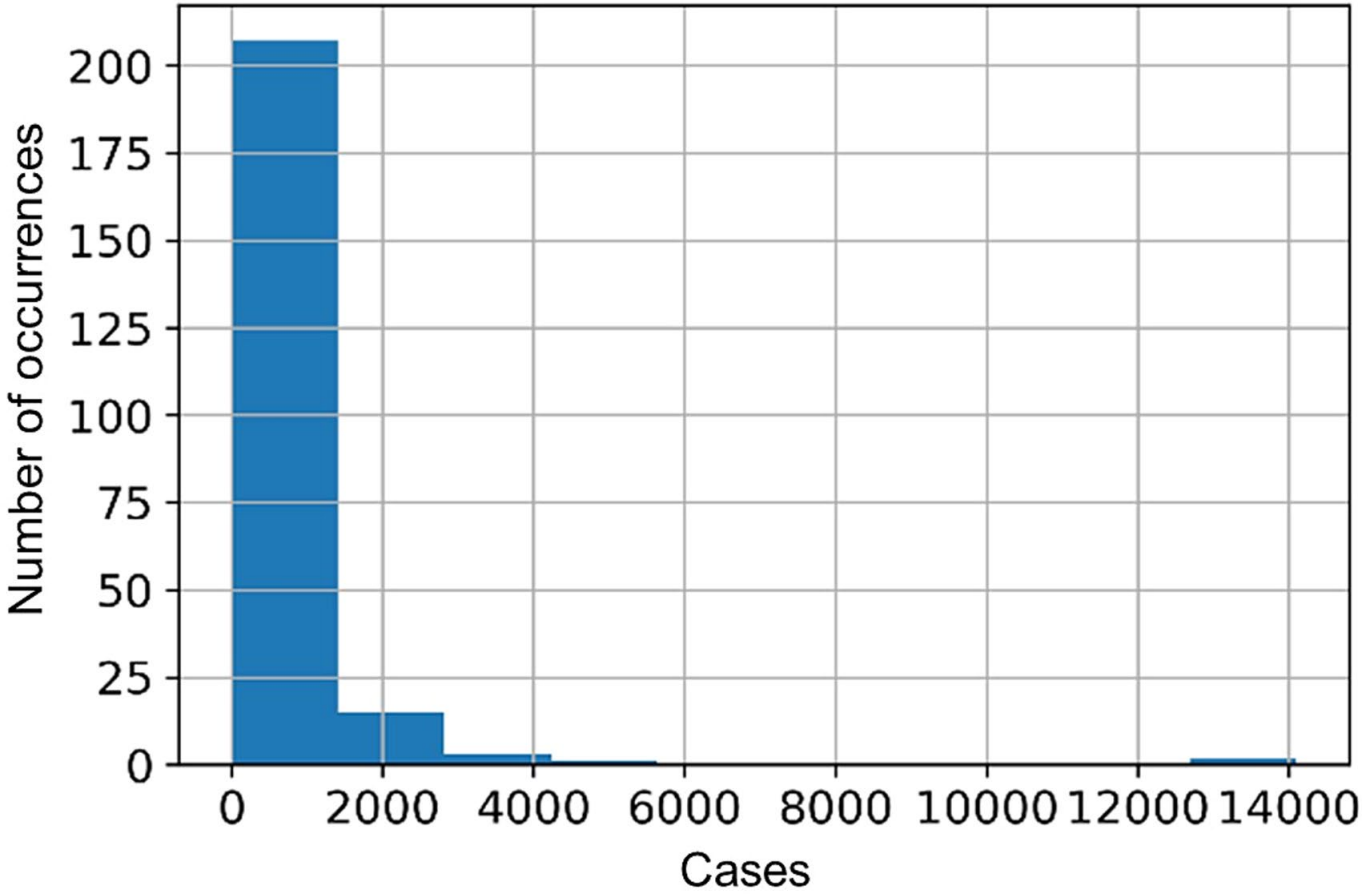
Histogram of time series of dengue cases in Recife from 2001 to 2019.

In this work, a Negative Binomial regression model was employed using the Python programming language, applying the Stats models library in version 0.13.1 (25). The choice of this method was based on the characteristics of the time series of dengue cases in Recife. The series data are of the discrete quantitative type and present overdispersion in the data distribution (Figure 3), indicating that the variance is greater than the average. These characteristics are necessary to apply the selected regression model (26, 41).

The Negative Binomial model used is type 2 (NB2), which means that the variance of the dependent variable is given by Equation 3:


(3) $$\sigma^{2}=\bar{X}+\alpha *{\bar{X}}^{2}$$

With *α* being the variance *σ* determination parameter (26). To obtain the same, Cameron and Trivedi (26) describe the use of auxiliary ordinary least squares regression without a constant (Equation 4):


(4) $$\frac{{\left(yi-\mu i\right)}^{2}-yi}{\mu i}=\alpha \mu i+ui$$

From this expression, *yi* would be the *i*-th value of dengue cases, *μi* the occurrence rate vector, characteristic for count data, and *μi* the associated error. In this way, it is possible to find the variance parameter *α* by isolating it from the expression, once the other terms are known and calculated in the software mentioned above.

The equation of the multivariate negative binomial regression model is defined by:


(5) $$y_{i}^{\left(r\right)}=\exp\left(\beta_{0}^{\left(r\right)}+\underset{p}{\sum }x_{p,i}^{\left(r\right)}\beta_{p,i}^{\left(r\right)}\right)$$

The model parameter *y_i_* is the number of dengue cases recorded in month or day *i*, *β*_0_ is the intercept and *x_p_* represents the explanatory variables, where *p* = 1, 2, 3, …, *n*; *β_p_* represents the levels of association between each meteorological variable and dengue cases.

Two simulations were conducted for each time series of dengue cases: the first with only meteorological data as explanatory variables, and the second with the addition of serotype count information, as shown in Table 3. In all simulations, by applying Equation 5, each variable is isolated, and the others are systematically varied to determine their degree of influence on the dependent variable (11, 39). By obtaining this information, the model predicts values of dengue cases chosen randomly throughout the time series.

**Table 3:** Variables used in the negative binomial model simulation.

Frequency	Simulation	Variables
Monthly	1	Insolation; precipitation; temperature; days of precipitation; pressure; relative humidity
Monthly	2	Insolation; precipitation; temperature; days of precipitation; pressure; relative humidity; DENV-1; DENV-2; DENV-3; DENV-4

Furthermore, the performance of the model was measured, in both simulations, by calculating the correlation of the predicted cases with the data of observed cases, also measuring the uncertainty of the forecast based on the average deviations of these same series as described by Lima et al. (11) showed in Equation 6.


(6) $$\mathrm{Deviatio}n_{\mathrm{predicted}}-\mathrm{Deviatio}n_{\mathrm{observed}}=\mathrm{Error}$$

To assess the evolution between the simulations, the standard deviations and the correlation coefficients between the predicted and observed case series were calculated as follows:


(7) $$R=\frac{\left(\frac{1}{N}\right)\sum_{n=1}^{N}\left(C_{p}-{\bar{C}}_{p}\right)\left(C_{o}-{\bar{C}}_{o}\right)}{\sigma C_{p}-\sigma C_{o}}$$

With *N* being the number of points in time or space, *C_p_* (*C_o_*) the predicted (observed) cases, ( ${\bar{C}}_{p}\left({\bar{C}}_{o}\right)$ ) the mean of the predicted (observed) cases and *σC_p_* (*σC_o_*) the standard deviation of the series of predicted (observed) cases.

## 3 Results and discussion

### 3.1 Observation data

**Figure 4:**
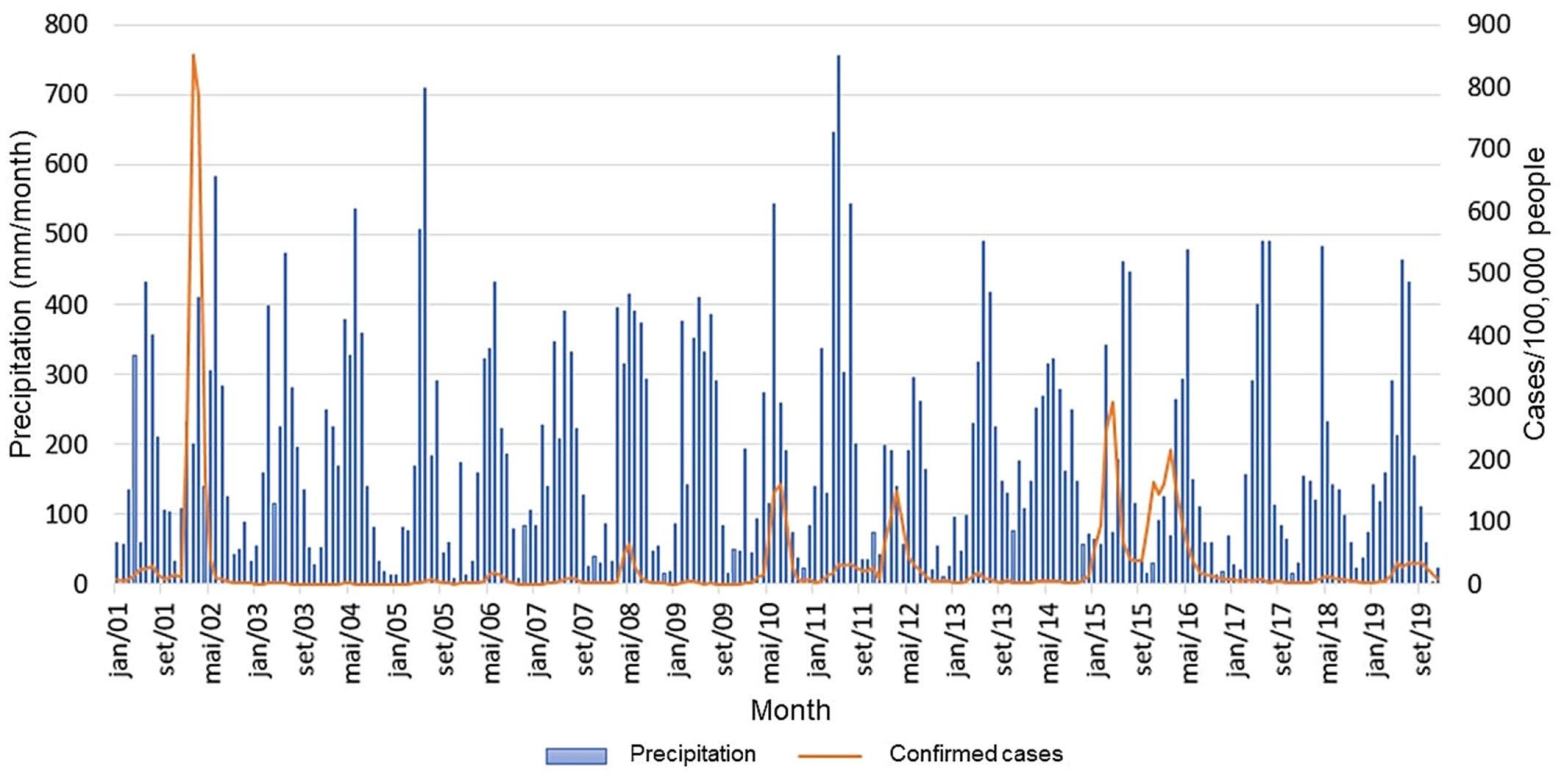
Time series of precipitation and confirmed cases of dengue per 100,000 people for the city of Recife from 2001 to 2019.

Figure 4 shows the comparison of the two time series. From this, it is possible to observe that dengue cases do not follow a well-established pattern compared to precipitation. There are periods with high peaks of cases (e.g., 2002, 2008, 2010, 2012, 2015, and 2016) that do not correspondingly match the peaks of monthly precipitation. Additionally, dengue cases do not present a defined seasonal pattern throughout the time series. The same is not observed for precipitation, whose monthly records show wave behaviour representing seasonal patterns.

A large epidemic period of dengue cases was observed in 2002. This year was characterized by significant epidemiological outbreaks throughout Brazil. For example, in 2001, the DENV-3 serotype was reintroduced in the country (4, 5). In this year, it is possible to suggest that the surge in cases is solely attributed to favourable weather conditions for vector proliferation, but also to the introduction of a new serotype into a previously unexposed population. The dynamic of 2010 can also be explained by this phenomenon as it marks the year when DENV-4 reemerged in some regions of Brazil after decades (27, 28). However, in the study area, the DENV-1 and DENV-2 serotypes were more prevalent (28). In 2015, however, the dominance of DENV-4 in Recife may explain the exponential increase in cases observed that year (29), making it the second significant epidemiological year in the time series.

Although there is a lack of data about serotypes (8), the years 2008, 2012 and 2016 also exhibited similar dynamics to those of other epidemic years, but without the introduction of new serotypes. Instead, the co-circulation of existing serotypes may have contributed to the peaks in dengue cases. While the absence of specific serotype data prevents definitive confirmation, it suggests that such dynamics are plausible. In addition, some years with high monthly rainfall did not have coincide with significant outbreaks of dengue. For instance, in 2005 and 2011, despite high rates of monthly precipitation, there were relatively few confirmed dengue cases with monthly maximums of 113 cases in 2005 and 515 cases respectively, which are much lower when compared to other epidemic years, such as 2010 which saw 2,677 just 1 month.

A possible factor that may also influence dengue cases is climate variability. One of the most important phenomena affecting South America and the study region is the El Niño Southern Oscillation (ENSO). Approaches investigating the relationship between dengue case time series and ENSO variability have shown considerable promise. Overall, a correspondence has been identified between major epidemics in the Americas and the periodicity phase of ENSO, with strong agreement in the 2 to 3-year period isolated from both time series (30, 40, 31). In Recife, ENSO positively affects precipitation, primarily during its negative phase (La Niña), which occurs due to the zonal displacement of the Walker circulation. The ascending branch of this atmospheric circulation is present over the study region, favouring increased precipitation when La Niña is active (32).

In Recife, the epidemiological years 2002, 2010, and 2015 coincided with El Niño events, which are known to be exceptionally favourable for the transmission of vector-borne diseases (31, 33). Although the positive phase of ENSO is associated with negative precipitation anomalies in the study region, these phenomena may also be linked to other weather variables, such as temperature, which also influences vector populations. Gonzalez et al. (40) demonstrated that El Niño events are correlated with dengue outbreaks in Venezuela due to the high incidence of positive temperature anomalies associated with the ENSO phase.

**Figure 5:**
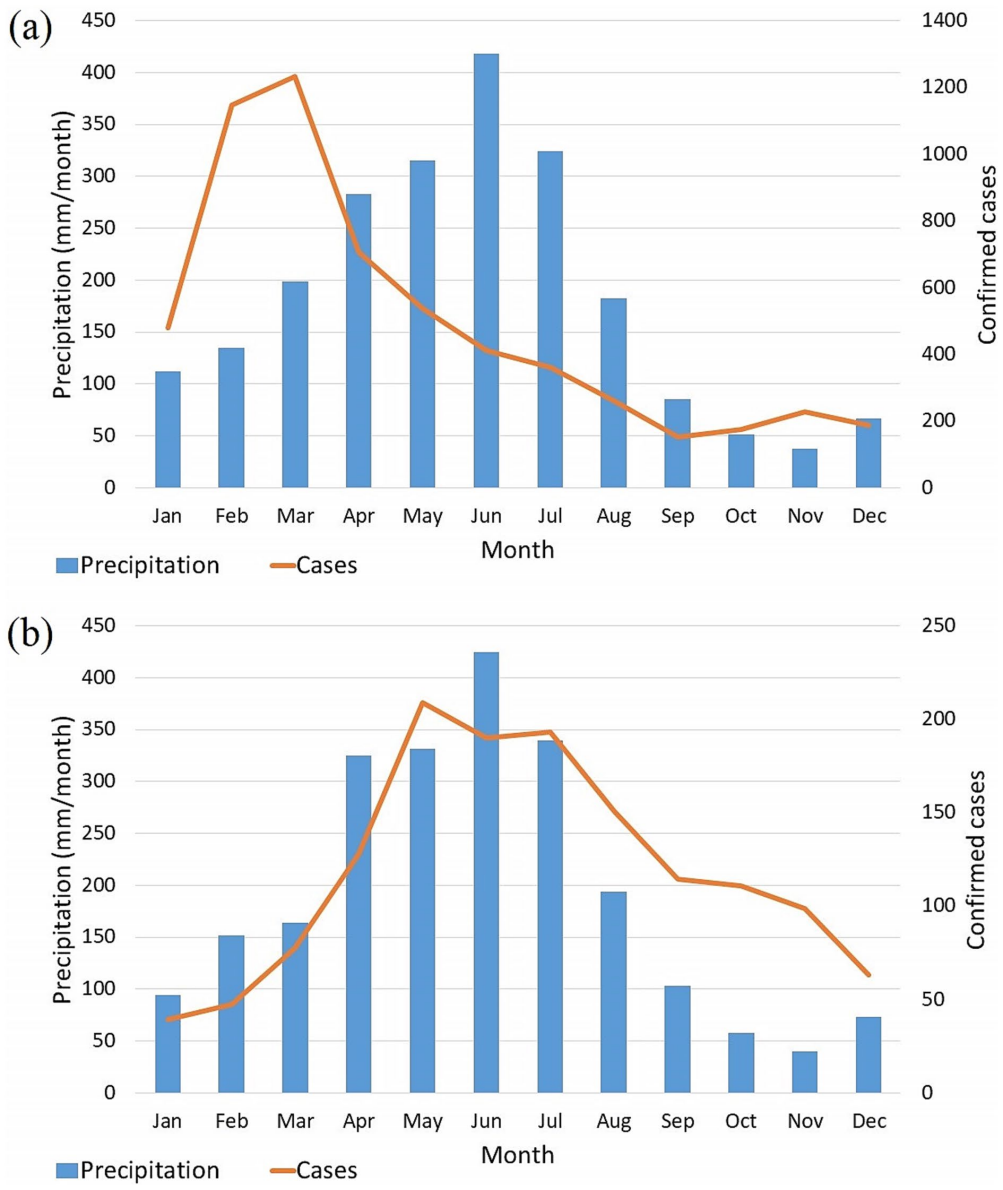
Monthly mean of Recife for confirmed cases of dengue (orange) and precipitation (blue). (a) Monthly means from 2001 to 2019. (b) Monthly means without the epidemiological years of 2002, 2008, 2010, 2012, 2015, and 2016.

Despite the discrepancies between the two time series, the periods with a significant increase in dengue cases coincide with the beginning of the rainy season in the region. This statement is complemented in Figure 5, which presents a correspondence with the periods of maximum occurrence between the two variables. For dengue cases, the peak is in March, April, and May (Figure 5a), and for rainfall, it is in May, June, and July. Similarly, it is also observed that the months with the lowest occurrence of precipitation are also the months with the lowest incidence of cases, i.e., September, October, November, and December.

Considering the possibility that the epidemiological events of the years 2002, 2008, 2010, 2012, 2015, and 2016 were mostly caused by the influence of the circulation of serotypes, these years were removed from the time series, and the monthly averages were recalculated. This step made it possible to observe a greater correspondence with the months of maximum and minimum between the two variables (Figure 5b), aligning the periods of case increases with the arrival of the rainy season (i.e., March, April, and May) and the decline of cases with the dry season (i.e., August, September, and October).

By removing the epidemiological years from the analysis and comparing the two patterns of observed monthly averages, it is possible to hypothesize that the count of dengue cases may be influenced by two factors. First, weather conditions, which are subject to seasonal variations, contribute to the persistence of the disease through the supply of water in containers and reservoirs suitable for the breeding of the mosquito. Secondly, the serotypes present in the population may explain the outbreak of cases independently of the season. The introduction of a new serotype or the interrelationship between those already present has the potential to cause much more expressive increases in cases, not following seasonal patterns. Evidence for this hypothesis is observed in the years 2002 and 2015, which show a significant increase in cases during the months of January and February, periods characterized by some of the lowest rainfall rates, alongside the occurrence of monthly cases. A similar pattern was also observed by Conde-Gutierrez et al. (9), who identified that during the dengue outbreak of 2022 in Mexico, the annual mean precipitation and minimum temperature were lower than in the year preceding the outbreak, not indicating a direct correlation. Furthermore, they identified that the increase in cases for that year occurred after precipitation and temperature reached their maximum values.

The first factor is more likely to be predicted than the second, as it is linked to weather patterns that can be described and predicted concerning the systems that cause precipitation in the study region. The second factor, on the other hand, is much more difficult to describe and evaluate since information on serotypes is not abundant and collected automatically in a unified way, unlike information on weather and climate. Furthermore, the true impact and potential dangers that the co-circulation of different types of dengue can cause are not well understood (2), making the construction of a prognostic tool for this factor even more complex. It is important to note that while the behaviour of the time series regarding dengue cases may be attributed to the two factors mentioned earlier, these factors are not independent of each other. In reality, the outbreaks triggered by the second factor only reach alarming levels when there is a substantial population of disease vectors. This, in turn, hinges on the presence of water in reservoirs and containers, which are primarily influenced by the first factor. Therefore, the effective control and elimination of potential breeding sites are crucial in preventing these outbreaks.

**Figure 6:**
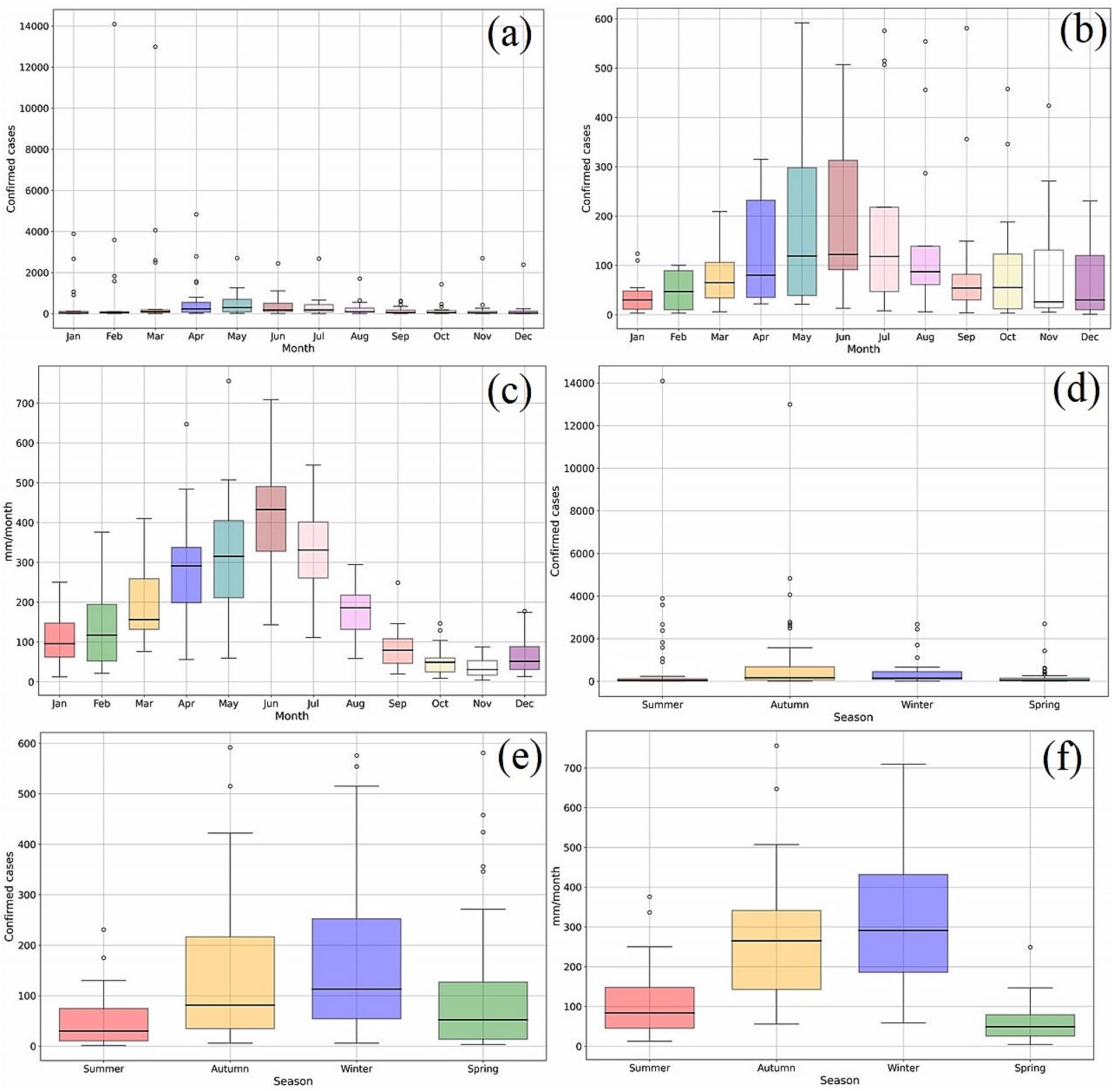
Box diagrams of the time series of dengue and precipitation in Recife from 2001 to 2019 and with the mean calculated after removing the epidemiological years of 2002, 2008, 2010, 2012, 2015, and 2016. (a) Monthly mean of confirmed dengue cases from 2001 to 2019; (b) Monthly mean of confirmed dengue cases without epidemiological years; (c) Monthly mean of precipitation from 2001 to 2019; (d) Seasonal mean of confirmed dengue cases from 2001 to 2019; (e) Seasonal mean of confirmed dengue cases without epidemiological years; (f) Seasonal mean of precipitation from 2001 to 2019.

Figures 6a,c shows that the months of April, May, June, and July have the highest values of maximum in the two variables analysed. This similarity is repeated for the months with the lowest incidence of cases in the months of October, November, December, and January.

The analysis excluding the epidemic years (Figure 6b) exhibits a similar trend in terms of monthly fluctuations compared to the analysis that includes these years. The latter, which encompasses the epidemiological years, notably display the most significant outliers. These outliers represent the epidemiological periods that diverge from the pattern associated with the first factor discussed earlier.

The same correspondence can be observed when analysing the data by season (Figures 6d–f), with the highest values in the series for both variables occurring in autumn (i.e., March, April, and May—MAM) and winter (i.e., June, July, and August—JJA). Nevertheless, the lowest values are found in the summer (i.e., December, January, and February—DJF) and spring (i.e., September, October, and November—SON) seasons.

Analogously to the analysis of monthly averages, Figure 6 also shows that explosive epidemics, such as the one that occurred in 2002, occur in such a way that the conjunction of epidemiological and health factors (i.e., influence of serotypes), act to dilute the power of statistical correlation with weather variables. This epidemic is indicated by the outlier of 14,000 cases, as shown in Figure 6d, representing an incidence rate of 852 new cases per 100,000 inhabitants in just 1 month, February, occurring in the summer whose mean values for both have the lowest rates of precipitation and cases (Figures 6e,f, respectively).

This outcome substantiates the absence of a direct correlation between weather conditions and sudden explosive epidemics. Such a correlation would manifest through outliers, signifying instances where these explosive episodes transpire during months characterized by heavy rainfall and a substantial surge in case numbers. In the context of the study in question, these outliers would correspond to autumn and winter, despite the conventional expectation that epidemics would be more likely to occur under favourable weather conditions during these seasons.

### 3.2 Disease cycle estimation

**Figure 7:**
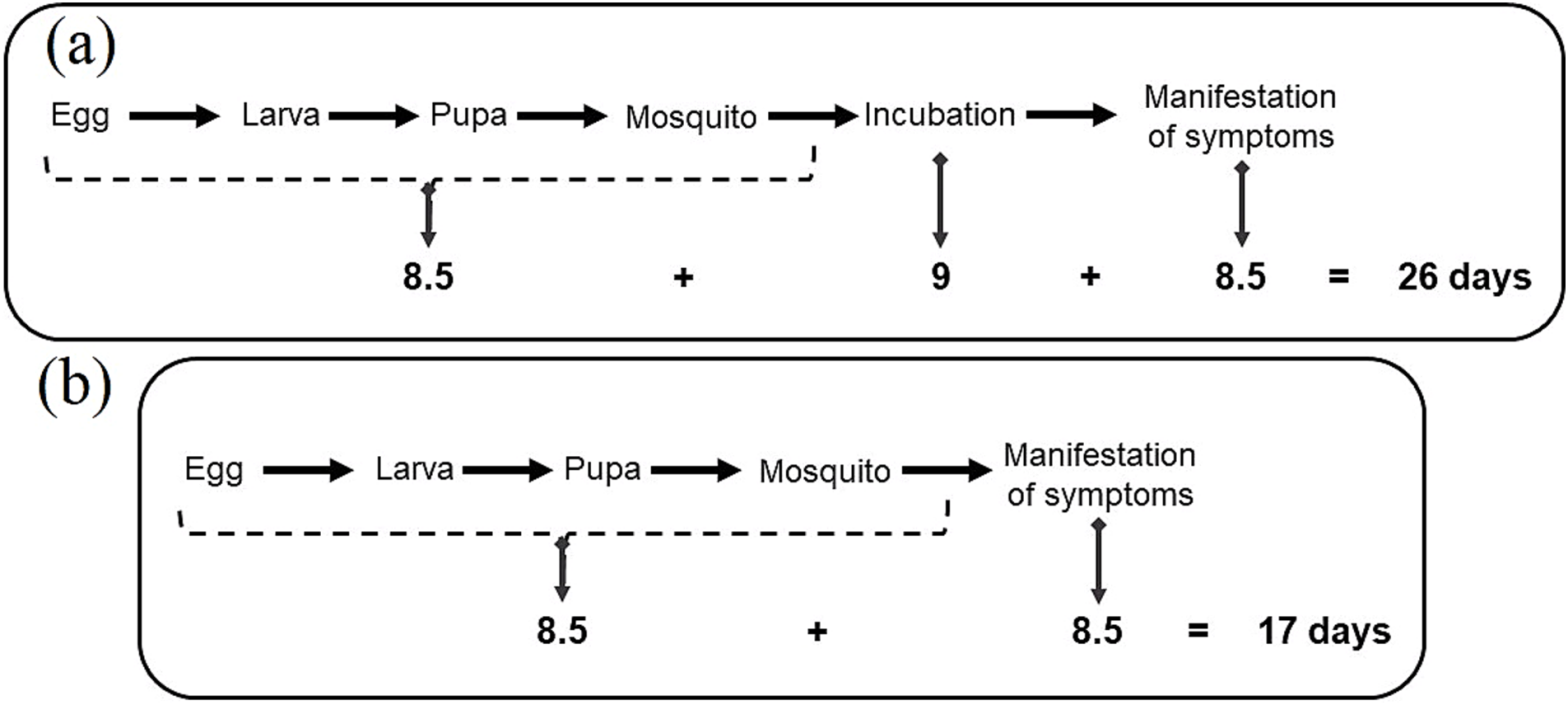
Scheme of the *Aedes aegypti* life cycle and transmission of dengue virus with average duration for each period in days (a) and (b) in case of transovarian viral transmission.

From the phases of the dengue transmission cycle, mean duration values were searched in the literature for each part of the cycle. This step is important for estimating the value of days to be used in the analysis of the epidemic years. The period of 7–10 days is found for the egg hatching to the mosquito adult phase (FIOCRUZ), 8–10 days corresponding to the infestation of the virus in the female mosquito and incubation to infestation (34) and 3–14 days of incubation until symptoms appear in an infected human (34). From these estimated values, the average values were selected for each period of each stretch indicated in the cycle and the total number of days was added, reaching a value of approximately 26 days of duration (Figure 7).

It is important to highlight that, concerning *A. aegypti*, there is evidence of transovarian viral transmission from infected females to newly generated offspring, as documented by Teixeira et al. (5) and Leandro (35). This means that the entire incubation and infection process within adult mosquitoes, which occurs when they feed on infected humans, can be omitted and the estimated period can be reduced from 26 days to 17 days, as new larvae are already carrying the virus (Figure 7b).

This value makes it possible to estimate the time between the creation of the larval proliferation environment, provided by the precipitation, and the manifestation of symptoms in the patient who sought a health unit that recorded the occurrence of the case. Thus, within this range of days, it is important to consider that the increase in cases in the respective month may be linked to the increase in vectors provided by a precipitation event that occurred in a period of approximately 26 days, which could be either in the month of occurrence as in the previous one. It is important to highlight that dengue is a disease in which the majority of cases are asymptomatic to mild (7), causing large part of those infected not to seek a health unit because they do not have symptoms. Therefore, it is important to consider a high degree of underestimation in the values recorded on public health platforms, not due to inefficiency in collection or lack of units, but due to the disease clinic itself.

### 3.3 Precipitation categorized

**Figure 8:**
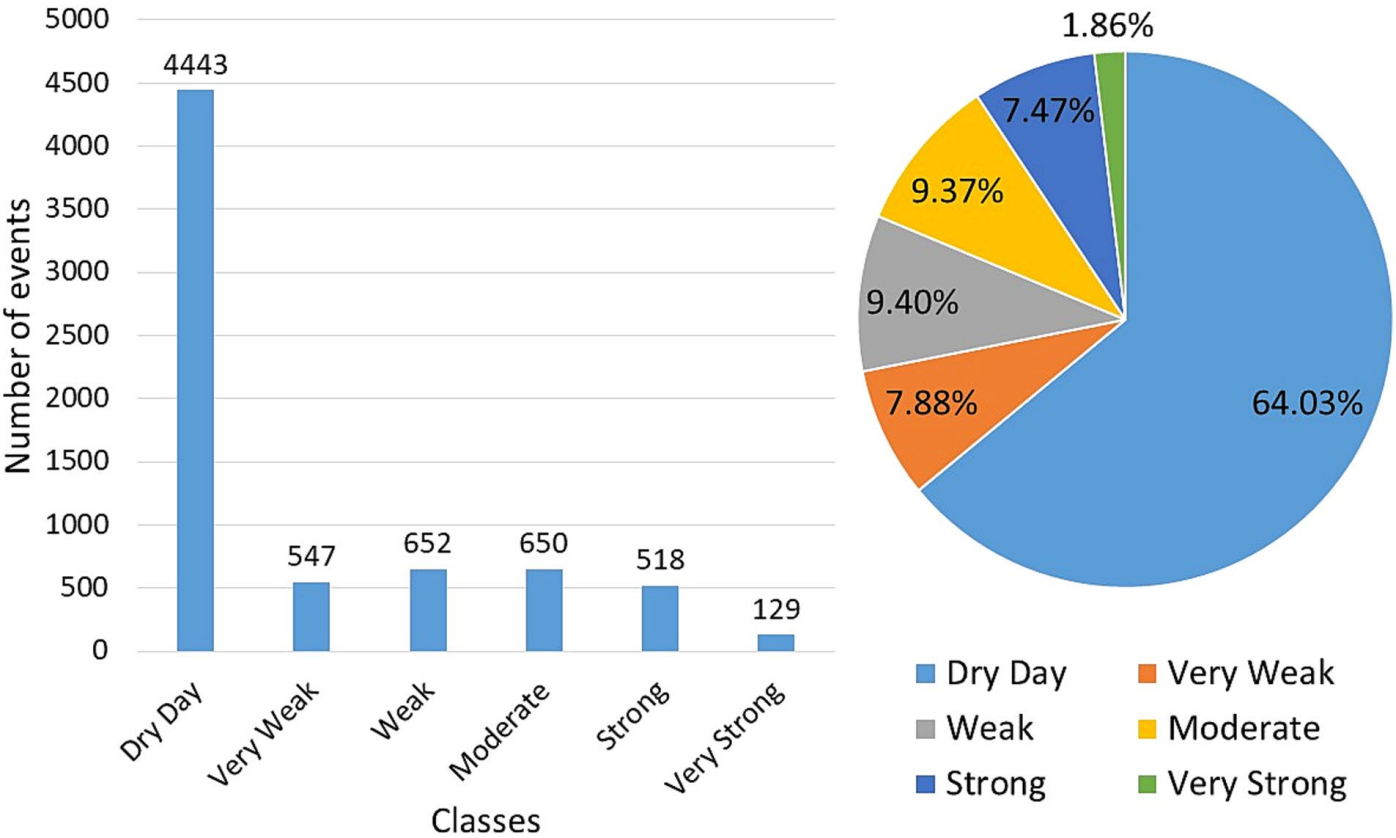
Distribution of classes of the 2001–2019 daily precipitation time series in Recife adopting quantile analysis, indicating the city’s precipitation profile along with the percentage of occurrences for each class by the total number of days in the series.

The values used to define the daily precipitation classes, obtained through quantile analysis, can be found in Section 4 of the Supplementary Table S1. Based on these classes, it is possible to determine the distribution profile of precipitation types in the city, as shown in Figure 8. The majority of the time series is represented by the ‘dry day’ class, characterized by daily precipitation values below 2.4 mm/day, accounting for 64% of occurrences in the time series, followed by weak precipitation (9.40%) and moderate precipitation (9.37%). The classifications of very weak and strong precipitation comprise 15.35% of the time series (7.88 and 7.47%, respectively), while the final classification, very strong precipitation, accounts for 1.86%.

Understanding these thresholds is important for other sectors of society, such as Civil Defence, which relies on this information for planning and managing different regions of the city that may be at risk (24). The general precipitation profile in Recife points to a predominance of dry days, as well as weak and moderate precipitation events. Such a pattern may be related to the geographic position of the study area, since it is located in a coastal region that receives daily influence from breeze events that cause precipitation according to the observed distribution. Additionally, the region also has the frequent operation of SASH that provides moisture transport throughout the year (13, 14). The position close to the Equator, which establishes little temperature variation throughout the year, along with these two systems, may be the main factors for maintaining precipitation in a weak and moderate regime. Strong and very strong precipitation events are likely linked to other systems that do not operate as consistently throughout the year as those mentioned above, and thus the rainfall associated with them is less frequent. Examples of these phenomena are the EWDs, which typically occur in winter (16, 36–38), and HLCVs in spring, autumn and summer, being more frequent in the latter (36).

**Figure 9:**
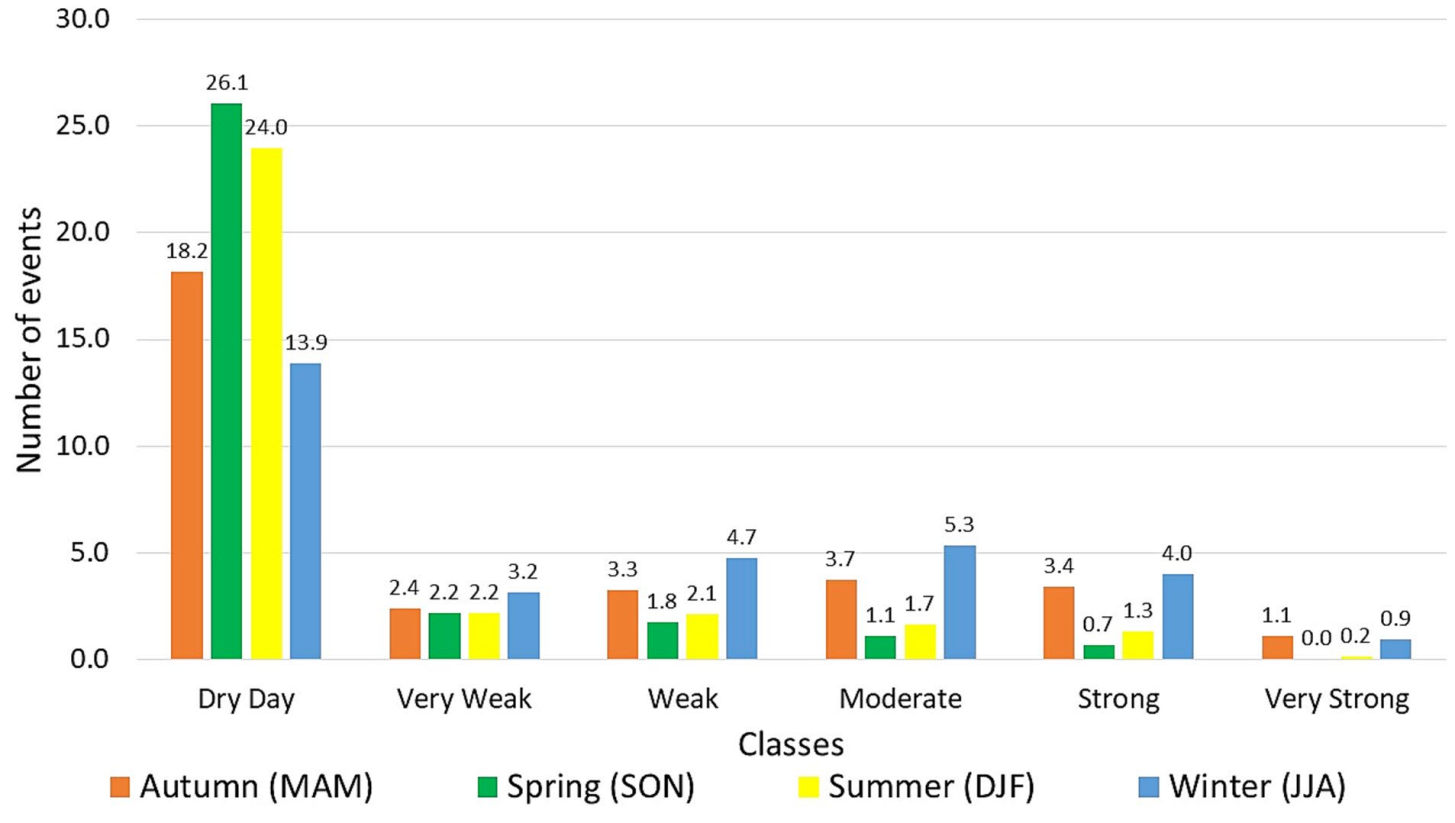
Distribution of the mean occurrence count of the daily precipitation classes from 2001 to 2019 for Recife considering the seasonal mean calculated in the four seasons of the year, being Autumn in the months of: March, April, and May; Spring: September, October, and November; Summer: December, January, and February; Winter: June, July, and August.

Analysing the profile of classes by seasons (Figure 9), we can see the relationship between the highest average number of occurrences of moderate, strong and very strong events with the months with the highest monthly rainfall in the autumn (MAM) and winter (SON). Both seasons have the lowest average number of dry days in the year and the highest averages in the subsequent classes, with winter representing the highest values, with the exception of very strong daily precipitation, which occurs more in autumn. Similarly, spring and summer, characterized by low monthly rainfall, have the highest incidence of dry days and low occurrence of subsequent classes.

As seen in Figures 5, 6, the average monthly peak of cases in the year occurs with the arrival of the rainy season from March to August (i.e., autumn and winter). Precipitation in this period is largely composed of the weak, moderate and strong classes since, compared to the dry season, an average difference of 3–4 days is seen to those classes. Thus, the higher incidence of these precipitation categories in the rainy season points largely to favour the formation and maintenance of *A. aegypti* breeding sites, consequently leading to an increase in the registration of cases, as observed in the respective period. Likewise, it is verified that the higher occurrence of dry days is related to the fall and low values of dengue cases. This relationship is observed in the spring and summer seasons, which signal large counts of dry days and a drop in case records.

**Figure 10:**
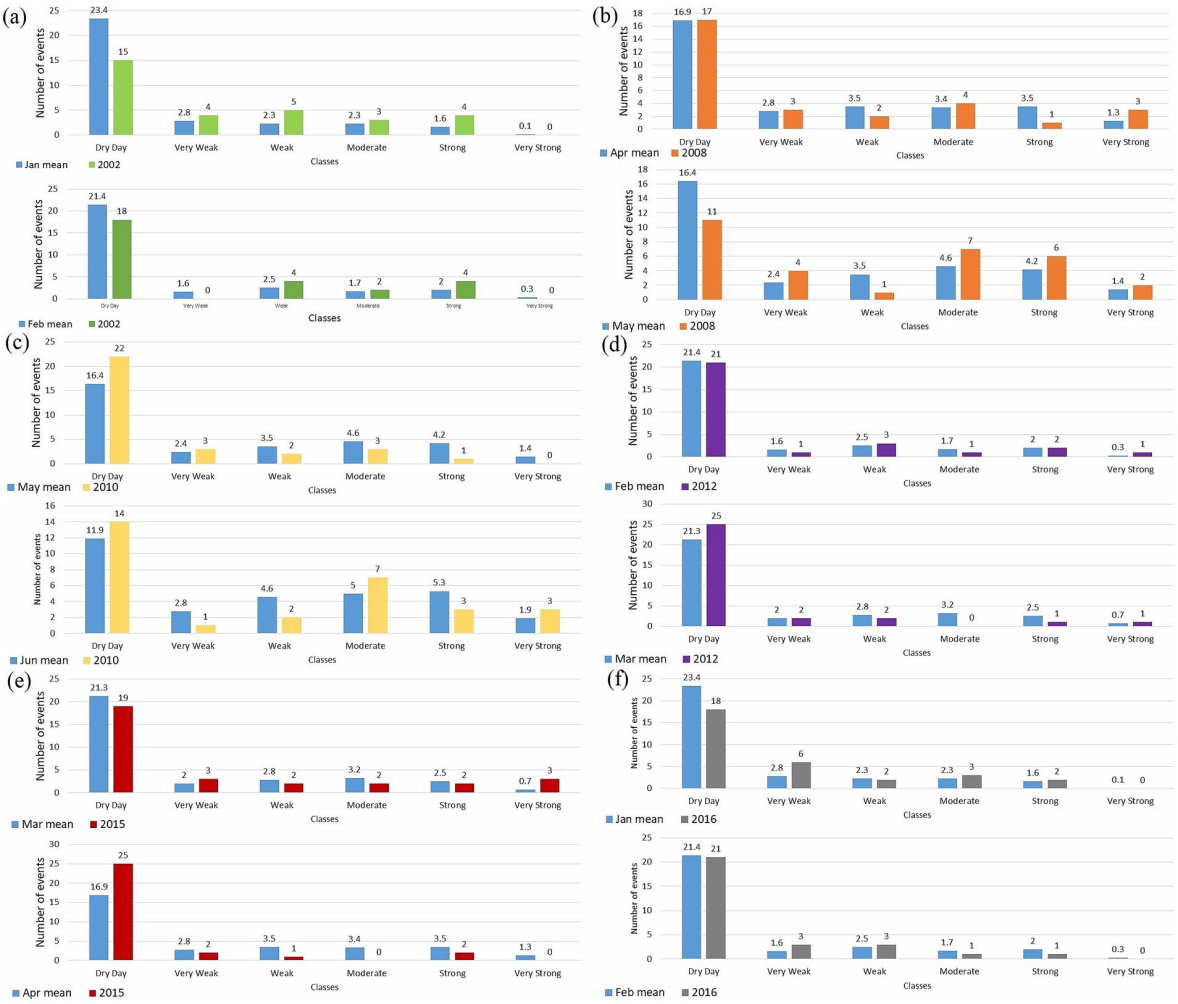
Comparison between the count of days in the daily precipitation classes for a given month with an explosive epidemic and the mean count of its respective month calculated over the entire time series from 2001 to 2019. (a) January and February of 2002; (b) April and May of 2008; (c) May and June of 2010; (d) January and February of 2012; (e) February and March of 2015; (f) January and February of 2016.

According to the study by Souza et al. (24), considering days with less than 2 mm/day of rainfall as dry is necessary, as this amount of precipitation is insignificant for studies evaluating water infiltration and retention in the soil. Such small amounts of water typically evaporate quickly due to the high temperatures prevalent year-round in the studied location. This is also present with regard to the breeding sites of the *A. aegypti* vector through the supply of water by precipitation, since dry days, considered in this study as values below 2.4 mm/day, presented an indirectly relationship with the dengue cases. In order to complement the analysis, the average count of precipitation classes was calculated for each month of the year and, with these values, these counts are compared with the aforementioned epidemic periods, referring to the years 2002, 2008, 2010, 2012, 2015, and 2016, as shown in Figure 10. With the knowledge of the disease cycle duration presented in Section 3.2, the month with the highest occurrence of cases and its predecessor month are chosen in the year of each epidemic.

Of the 6 years evaluated, four of them present their maximum number of dengue cases in months with an low rainfall average, these are the years 2002 (January and February), 2012 (February and March), 2015 (March) and 2016 (January and February). In January and February 2002, there was a lower record of dry days compared to the average for the respective months (Figure 10a), with emphasis on the 15 dry days recorded in January 2002, which are close to the June average of 11.9 days (Figure 10c), characteristic of few dry days and high rainfall. However, the lower occurrence of this class also shows a greater number of records for weak, moderate and heavy rainfall, with January 2002 being the month that most exceeded the average (Figure 10a).

As previously discussed, the year 2002 is marked by the introduction of the DENV-3 serotype to the national territory and is primarily responsible for the magnitude of cases recorded for this period. However, the profile of classes of daily precipitation found points to a greater likelihood of an increase in cases, as there was a lower incidence of dry days and greater for weak, moderate and strong precipitation compared to the average. Such factors may have influenced the expressive increase in cases, since, for diseases that depend on a transmission vector, such as dengue arbovirus, such epidemics would only be possible with the massive presence of vectors, which is due by the creation and maintenance of the proliferation environment provided by the weather conditions highlighted. This year experienced an El Niño event, but it cannot be linked to the dengue outbreak that occurred, as the El Niño began in the latter part of the year, according to the National Oceanic and Atmospheric Administration (NOAA), while the dengue outbreaks occurred in the first 2 months.

A similar pattern can be observed for May 2008 (Figure 10b). Despite being a typically wettest month when compared to February, there is a lower number of dry days than the average, with an excess of the same in the very weak, very strong, strong and moderate precipitation classes, with emphasis on the last two. Its predecessor month (Figure 10b) remained in the average for the dry days but presents the occurrence of three very strong events that, on the other hand, end up leaving the weak and strong classes below average. These months are at the beginning of the rainy season for Recife, making it more susceptible to accounting for very strong precipitation events that can provide a deviation from the average in the other classes. Similar to what was observed in the period of 2002, the creation of more suitable environments for the proliferation of vectors may be linked to a lower count of dry days and higher for moderate and heavy rainfall in May 2008. The events analysed in 2010 (Figure 10c) also occurred during the rainy season, specifically in May and June. However, both months experienced a higher number of dry days than their respective averages, with a significant increase above the average occurring only in June for the moderate and very strong classes. Additionally, 2010 was marked by an El Niño event during the outbreak months, which may explain the increased occurrence of dry days.

The 2012 and 2015 bimesters show counts close to the average with the exception of March 2015, which accounted for 3 very strong precipitation events and March 2012 with dry days above the average of the month. However, the subsequent classes do not significantly exceed the average and are even below for weak, moderate and strong classes for the month of February and March (Figures 10d,e). January 2016 (Figure 10f) has a lower number of dry days and a higher one for very weak rainfall, remaining practically on average in the other two-month classes.

From these last 4 years described, it is possible to conjecture that the distribution pattern of the classes did not interfere in a consistent way with the increases in cases, since dry days were recorded at the average or higher in the case of the bimester of 2010 and April of 2015. Other classes are on average or do not point to a large deviation that signals, at least in terms of precipitation, the high increases in cases in these periods.

The values of the quantile intervals obtained for the classification of monthly precipitation are showed in Section 4 of the Supplementary Table S2. From these intervals, the six epidemic periods separated in the previous analysis are highlighted in Table 4.

**Table 4:** Events of dengue epidemic peaks in Recife recorded between 2001 and 2019 with their proper precipitation classification in the month and case count.

Month/Year	Classification	Precipitation (mm/month)	Confirmed cases
Jan/2002	Rainy	231.6	3,886
Feb/2002	Normal	199.5	14,094
Apr/2008	Rainy	314.2	800
May/2008	Very rainy	415.7	1,090
May/2010	Normal	114.3	1,252
Jun/2010	Very rainy	543.9	2,442
Feb/2012	Normal	189.8	1,831
Mar/2012	Normal	138.8	2,495
Mar/2015	Rainy	341.2	4,059
Apr/2015	Dry	74.6	4,833
Jan/2016	Normal	124	2,670
Feb/2016	Dry	70	3,589

From the six bimesters evaluated, only two obtained the dry classification, being the month of April 2015 and February 2016. Both are presented as peak months of cases in the referring years and, nevertheless, are preceded by months with rainy classification (March 2015) and normal (January 2016). In addition, April 2015 has one of the highest values of cases in the time series, even though it is classified as a dry month. For the other periods, it is evidenced that either their respective peaks are preceded by a rainy or very rainy month, or they already have this classification. For example, February 2002, which is classified as normal, but is preceded by a rainy month. Still as an example, June 2010, which is defined as very rainy, preceded by May 2010, with normal monthly accumulated. The exception applies to 2012, which is normal in both months.

From this classification, one hypothesis can emerge by assuming that the years 2015 and 2016 are marked by the influence of the DENV-4 serotype over the weather conditions, since the classification of the monthly precipitation of these 2 months was the only one, of the six evaluated periods, which present dry months (i.e., February 2015 and 2016). Together with the analysis for the classes of daily precipitation, presenting themselves mostly within the average or even below it, as weak, moderate and strong precipitations.

Likewise can be said for the peak referring to 2002, which may be governed simultaneously by two factors: serotypes, represented by the introduction of DENV-3, whose immunity the population did not have until then, and weather, constituted by the rainfall classifications, showing a rainy month preceding the maximum number of cases and, nevertheless, anomalous values of the occurrence of the classes weak, moderate and strong for the bimester. The simultaneous action of these factors may be the cause of the episode having the highest magnitude of confirmed dengue cases of the entire time series.

It should be noted that 2002, 2015, and 2016 were the 3 years that had the highest monthly records of confirmed dengue cases, being discrepant in order to be recorded as outliers in the analysis of box diagrams of Figure 6. Such values may be reached by the aggravation of the insertion of new serotypes in the population, as already described. The remaining years, as they do not present this same particularity, show the values of the peaks of cases much smaller, even when measuring favourable precipitation rates for the explosion of the vector population. An example of this is the bimester formed by the months of April and May of 2008, which are classified as rainy and very rainy, respectively, but have two of the lowest values of cases recorded between the epidemiological periods evaluated. In this way, it may be possible to affirm that only the factor described by the dynamics of serotypes in the population has the potential to raise the case count to the levels observed in the years 2002 and 2015.

### 3.4 Regression model predictions and analysis

**Figure 11:**
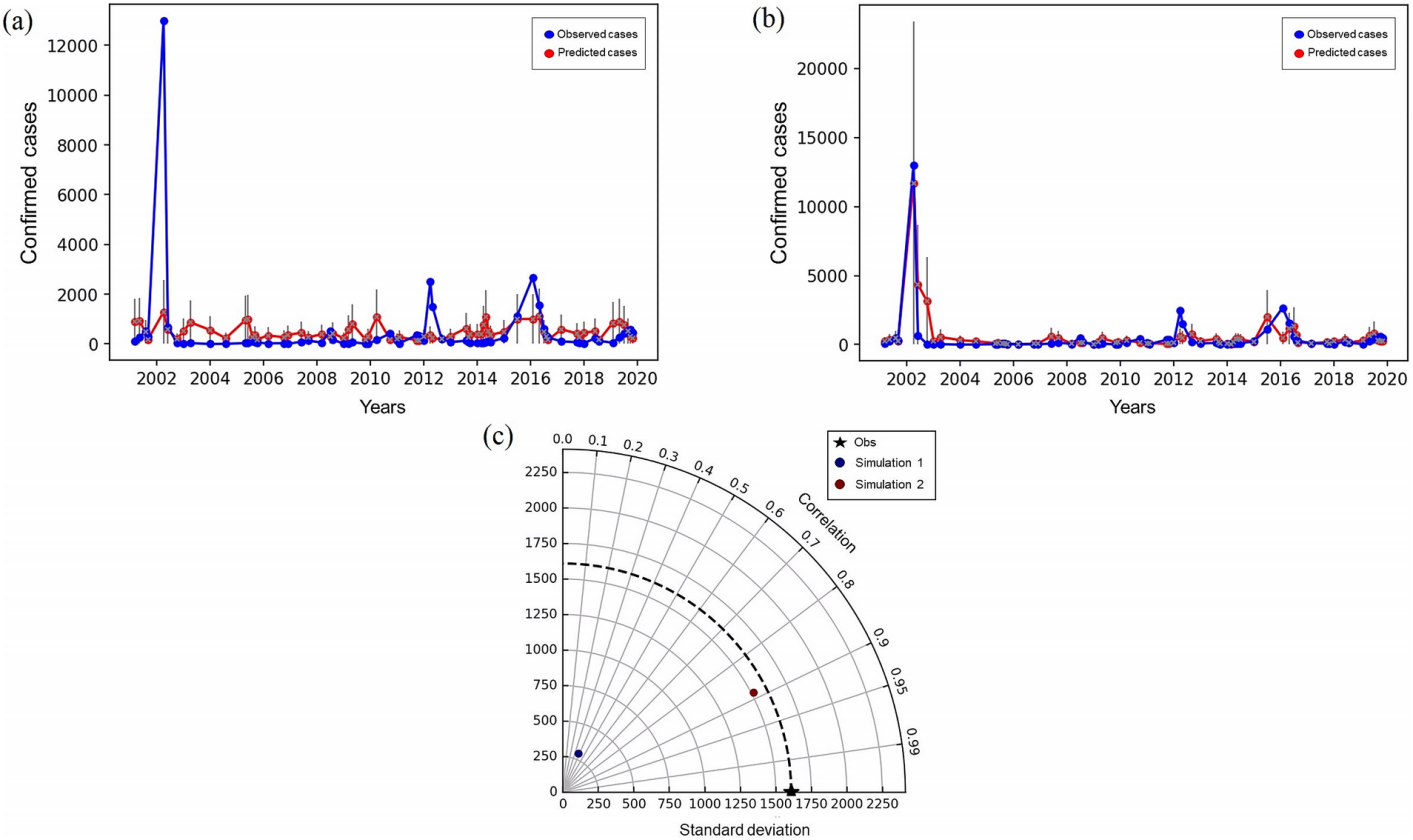
Regression analysis forecasts and results of monthly dengue cases for Recife from 2001 to 2019. (a) Simulation 1 considering as independent variables insolation, precipitation, temperature, days of precipitation, pressure and relative humidity. (b) Simulation 2 considering the same variables as the previous round with the addition of the percentage of DENV-1, DENV-2, DENV-3, and DENV-4 serotypes; (c) Taylor diagram of the time series generated from simulations 1 and 2 compared with observed data.

The predictions of dengue case counts made by the regression model are shown in Figure 11. After fitting the model, the program selects a set of test data from the observed series of counts and makes predictions for these values. For this reason, Figure 11 does not present all the peak periods of cases described in the previous analyses, highlighting only the epidemics of 2002, 2012, 2015, and 2016. The values of precipitation days, present in the monthly time series provided by the INMET conventional station in Recife, are replaced by the sum of the counts of days corresponding to the very weak, weak, moderate, strong, and very strong precipitation classes, as calculated in Section 3.3. This substitution is made to exclude precipitation values below 2.4 mm/day, classified as dry days, generating corrected values of precipitation days for each month.

Similar to the findings of Lima et al. (11), the predicted cases show an underestimation during peak periods and an overestimation during other months in simulation 1 (Figure 11a), indicating the model’s tendency to fail in representing disease peaks when using only meteorological data. As a result, the correlation (Equation 7) between the predicted and observed cases is calculated to be 0.37 (Figure 11c), demonstrating a suboptimal representation of the entire series. The only statistically significant independent variable for this first simulation is temperature, with a *p*-value of 0.03.

The calculated error is −350.1, where the negative sign indicates that the mean of the predicted cases is more representative than that of the observed cases. Mean deviation assesses data variability around the mean: greater variability results in a higher deviation, making the mean less representative. The observed cases exhibit a higher average deviation due to the peaks in the series, while this is not the case for the predicted cases in simulation 1, where these peaks are absent.

Performing the *χ*^2^ test (43), it is verified that its value for the calculated tests (*χ*^2^ = 145) is smaller than its critical value (*χ*^2^ = 182), considering a significance level of 95% (i.e., *p* < 0.05) and 153 degrees of freedom. Thus, the frequency distribution of the series of observed cases is different from that predicted by the model, showing that it was not able to adequately represent the observed cases of the disease.

As described in Section 2.4.1, the serotype with the meteorological data are added in simulation 2 as an explanatory variable (Figure 11b). The results show that a large part of the overestimation observed in the previous simulation is mitigated, as well as a peak in predicted cases is added referring to the 2002 epidemic, whose magnitude is considerably close to the observed. However, the other peaks of the years 2012 and 2016 are still not represented satisfactorily, remaining underestimated. Thus, the correlation found for both series presented a value of 0.88 (Figure 11c), a value much higher than the previous simulation of 0.37, demonstrating a better correlation between predicted and observed cases. The statistically significant variables were insolation, precipitation, DENV-1, DENV-2, DENV-3, and DENV-4.

Simulation 2 had an error of 22.5, much smaller compared to the previous simulation. This may be due to the decrease in overestimations and mainly to the signalling of peak of cases in 2002, which is the most discrepant peak for both the predicted and observed series, causing an increase in the mean deviation. The value of *χ*^2^ found for the predicted series is *χ*^2^ = 300, being higher than its critical value of *χ*^2^ = 178, considering the significance level of 95% (i.e., *p* < 0.05) and 149 degrees of freedom. This result indicates that the frequency distribution of the series of predicted cases is the same as that predicted by the model, and it is possible to affirm that there was a satisfactory representation of the cases observed in this second round of the model.

The evolution between the two simulations can be seen in Figure 11c, which shows not only the increase in the correlation between the predicted cases of the model and the observed ones, but also by the approximation of the standard deviation of the series generated with the data of reference.

The evaluation of error in the data regression process is crucial, given the specific characteristics of the data under analysis. This assessment involves scrutinizing the average deviation between predicted and observed dengue cases, offering valuable insights into the alignment of averages in both data sets. The presence of past epidemic peaks can influence the mean deviation value. When the error approaches or is very close to 0, it signifies that the means in both data series are closely representative of each other. This indicates that the regression of confirmed dengue cases effectively captures deviations from the mean, particularly when considering epidemic peaks as observed values.

## 4 Conclusion and future perspectives

### 4.1 Conclusion

The present study investigate the outbreaks of dengue occurred between 2001 and 2019 in Recife.

The analysis regarding weather and climate data shows a distinguish relationship between the dengue annual cycle and the rainy and dry seasons of the city. The classified precipitation also indicate a correlation between the rainiest periods and the peak in case numbers. A higher occurrence is observed during seasons with a greater number of days characterised by weak, moderate, and strong precipitation, while a lower occurrence is noted in seasons with more dry days. However, it is identified that this relationship is not entirely straightforward or deterministic.

The analysis reveals notable discrepancies, particularly regarding the outbreaks. Most of the identified epidemiological periods occurred outside the expected rainy season, as seen in 2015 and 2016 when severe epidemics transpired during the dry season and in months classified as dry by the quantile analysis. Moreover, 2015 coincided with an El Niño episode, which is characterized by reduced precipitation in the study area. A similar pattern was observed in four out of the six identified epidemiological periods, where outbreaks occurred in typically drier months for Recife, such as January and February. Likewise, the 2-month period of 2008 demonstrated that even during traditionally rainy months, this condition alone is not enough to create an extreme outbreak.

These findings underscore the multifaceted nature of the interaction between precipitation and dengue incidence that is also showed by the literature. While rainfall plays a significant role in dengue dynamics, other contributing factors and complex dynamics are also at play, emphasizing the need for a more comprehensive understanding of the underlying mechanisms driving dengue outbreaks in the city.

Consideration of the influence of circulating serotypes within the population is crucial for accurately representation and justifying the identified epidemic periods. This information might explain the outbreaks that only weather conditions fail to explain. When combined with the last, these serotypes emerge as a pivotal variable contributing to the explosive rise in cases. Their inclusion significantly enhances the correlations between the initial and subsequent simulations of the monthly frequency data, further reinforcing their importance in the recommended regression model.

It is important to highlight that a single database was not found that provided information on serotype counts in the population for the entire period of analysis considered by this research (i.e., 2001–2019). As a result, it was required to search for other sources in addition to those provided by the public platform DATA-SUS health system. This consideration holds significance because, while the inclusion of these available data has notably enhanced the representation of the dengue case series, a more extensive coverage of these data during the missing periods could yield even more promising results.

The discrepancies observed in the time series analysis, especially the mismatch between substantial peaks in precipitation and the absence of corresponding dengue cases (e.g., in 2005), may be explained by government-led initiatives to combat *A. aegypti*. These measures, which involved extensive mosquito control campaigns, including home inspections by public agents and the application of insecticides, potentially acted as a deterrent to dengue epidemics during years with favourable weather conditions.

Nonetheless, it is essential to note that no concrete data or information could be found to quantify the exact impact of these measures. Consequently, we cannot definitively ascertain the predominance or effectiveness of the mitigation actions taken by public policies during the study period. The precise influence of these interventions on the observed dengue patterns remains uncertain and warrants further investigation.

### 4.2 Perspectives for future work

The significance of information on the prevalence of serotypes in the population cannot be overstated when it comes to representing and predicting the most intense dengue outbreaks. Expanding the coverage and frequency of testing them daily can enhance the performance of regression analysis at this temporal scale and provide more detailed insights for monthly analyses.

This study considered the city of Recife, a locality whose geographic position has little seasonal variation in weather variables such as temperature and relative humidity. A study using the same methods for a region whose weather conditions vary drastically throughout the year, such as southeastern Brazil, can serve to better understand the influence not only of precipitation, but also of temperature and humidity.

## Data Availability

The datasets presented in this study can be found in online repositories. The names of the repository/repositories and accession number(s) can be found in the article/Supplementary material.
