# Supplementary material for: Analysis of the interrelationship between precipitation and confirmed dengue cases in the city of Recife (Brazil) covering climate and public health information
Source: Front Public Health. 2024 Oct 23;12:1456043. doi: 10.3389/fpubh.2024.1456043 (PMC11537940; doi:10.3389/fpubh.2024.1456043)
Supplement: Supplementary file 1 [file Data_Sheet_1.docx]

**Analysis of the interrelationship between precipitation and confirmed dengue cases in the city of Recife (Brazil) covering climate, sanitation and public health information**

**Supplementary Material**

**Section 1 – Daily data**

For this work, daily frequency data were also obtained through the Notifiable Diseases Information System (SINAN in Portuguese), which, in turn, is available from 2013 to 2019 and, again, refers to the patient’s municipality of residence for Recife, in order to reduce the allochthonous cases. Although the information is available, the authors decided to include this analysis in a supplementary material section due to several problems associated with the different frequency of the data available, as there is no dataset for serotypes at a daily frequency. As a matter of curiosity, the same methods used in the monthly analysis were applied to this information.

For the simulations of daily cases (Fig. S1), an undulating pattern is observed in the series of predicted cases (Fig. S1a) that is mitigated by the insertion of serotype information in the simulation (Fig. S1b). As observed for the monthly data, simulation 1 indicates an overestimation in most of the forecasts, with the exception of the observed peaks in 2015 and 2016, which are underestimated. With the addition of monthly serotype information, the pattern is modified, and there is signaling in these 2 years.


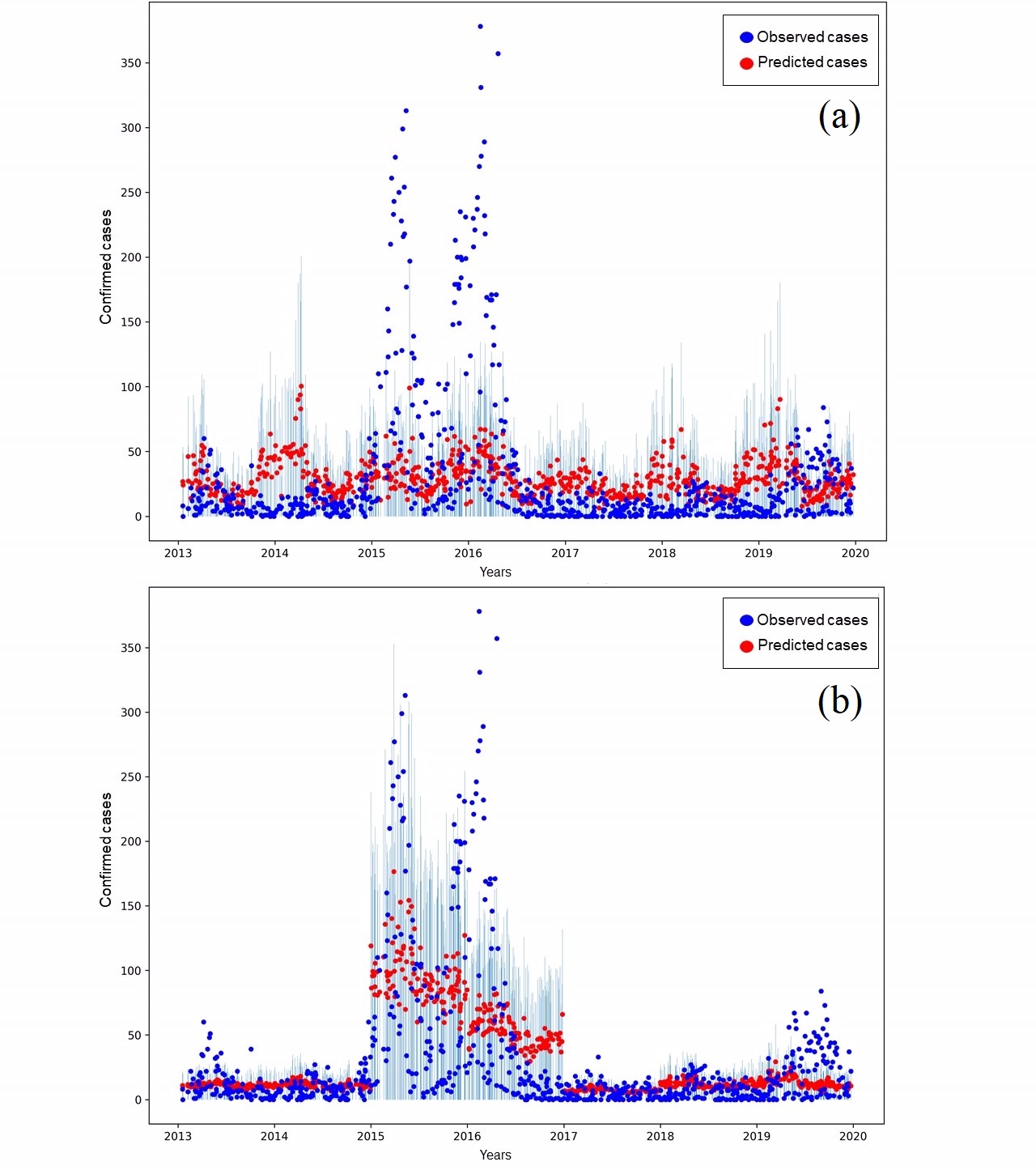


**Fig. S1**Forecasts of daily dengue cases for the city of Recife from 2013 to 2019 through non-binomial multivariate regression **(a)** Simulation 1 considering as independent variables insolation, precipitation, temperature, pressure, relative humidity, minimum relative humidity **(b)** Simulation 2 considering the same variables as the previous round with the addition of the percentage of DENV-1, DENV-2, DENV-3 and DENV-4 serotypes

However, due to the much larger number of data and the application of serotype counts in the monthly frequency, as it is not available in the daily, both simulations cannot satisfactorily represent the distributions present in the series of daily cases, calculating values of correlation less than satisfactory (Fig. S2), with the first reaching the value of 0.22 and the second 0.59.


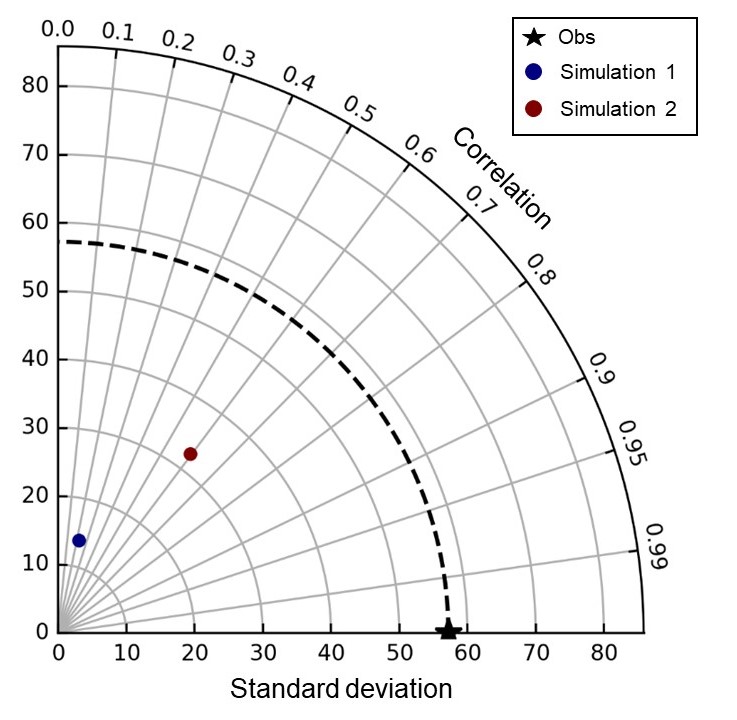


**Fig. S2** Taylor diagram of the time series generated to forecast daily dengue cases in the city of Recife for simulations 1 and 2 compared with the values observed in the health posts

The errors calculated for simulations 1 and 2 are presented at values of -24.6 and -8.6, respectively, showing a decrease between them. Although the numbers are smaller than those recorded for the simulations with monthly data, such magnitudes are expressed by the nature of the data to be validated, so that the values of daily records will always be smaller than those accumulated monthly, resulting, therefore, in different magnitudes for their respective averages and, nevertheless, for the average deviations.

Therefore, the calculated error of 22.5 in the second simulation for the accumulated monthly cases is much more significant as it comes from 2 data sets whose averages for the predicted and observed cases are 589.1 and 481.5, respectively. Meanwhile, for the daily cases, the average values are much smaller, with the average of 30.1 and 31.6 for predicted and observed cases, respectively, reflecting the magnitude found in the error.

The daily analysis of data in the regression model proved to be difficult to represent due to the characteristics of the time series of confirmed dengue data, which had several null values that were not predicted at any time in both rounds performed. This problem can be circumvented by analyzing the daily data by the ones accumulated in epidemiological weeks, without, therefore, these null values are difficult to represent.

**Section 2 – Regression Models**

It is necessary to understand the relationship between variables and how they influence each other. One way to do this is by using what is called regression analysis. Through a mathematical expression, it is possible to estimate a value of a dependent variable y being governed by other n independent variables X, observing which of these variables is the most significant before the same dependent variable. Although initially, there is no causal relationship between the variables analyzed, for example, cases of dengue and precipitation in the municipality, it is possible to make this estimate using this method (Hoffmann, 2015).

Generally speaking, regression analysis can be expressed by:

Y=f(x1,x2,x3,…,xn) (S1)

Where Y being the independent variable which we wish to predict, f is a function described by the independent or explanatory variables X_n_ (n = 1, 2, ..., n).

The characteristics of the dependent variable and the time series will determine the type of analysis and regression model to be used, as there is no single method for application. For example, in case the variables present a behavior that approaches a linear function, simple linear or multivariate regression is applied, depending on the amount of explanatory variables available. The characteristic of the dependent variable itself has a weight, which can be quantitative (discrete or continuous) or qualitative, and for each case, there are different methods for performing a regression analysis.

Despite being a widely studied area of statistics, its application in meteorology and health is still relatively recent. For example, Lima et al. (2008) made use of this method in order to try to predict the count of dengue cases in the states of Alagoas and Paraíba with information regarding the thermal conditions of the Atlantic Ocean, Southern Oscillation index, and precipitation through a linear regression multiple. Although satisfactory results were found for the prediction of cases (both spatially and temporally), the use of a linear model resulted in some inaccuracies derived from the limitations of the method, raising the hypothesis that the application of a non-linear model improves the forecasts.

**Section 3 – Sanitation and Land use**

For a spatial visualization of the basic sanitation conditions that favour the creation of mosquito breeding sites, data from the last 2010 Census of the Brazilian Institute of Geography and Statistics (IBGE in Portuguese) were obtained. The data refer to the number of households in each municipality in Brazil that use a certain type of basic sanitation. For proper mapping, the percentage of this information is calculated in relation to the total number of households present in the respective municipalities.

Land use data were also obtained from the year 2018 for the state of Pernambuco from the product generated by the IBGE entitled Monitoring the Coverage and Use of Land in Brazil. This product uses satellite images to compose a map that has statistical grids of 1 km² throughout the national territory, providing information on coverage classes identified in the area, such as pasture with management, vegetation, forests, artificial areas, etc. (IBGE, 2020).

Data on confirmed cases of dengue in each municipality were obtained through the public platform DATA-SUS, made available by the Ministry of Health, for the years 2001 to 2019 on a monthly time basis. Notification data refer to dengue cases for patients residing within the reporting municipality. With these data, the accumulated 18 years were made in order to identify the most vulnerable municipalities in epidemics and in annual cycles, calculating, for this purpose, the rate of cases per 10 thousand inhabitants to make it possible to compare municipalities with different populations (Bonita et al., 2010; São Paulo, 2022). Information on basic sanitation conditions was also obtained by the DATA-SUS platform, but with data from the IBGE itself from the last national census carried out in 2010, with the exception of land use maps, which surveys are carried out every 2 years, the last one available in the period of analysis of this work in the year 2018.

Both types of water supply evaluated in Fig. S3 are practically not used in Recife or in neighbouring municipalities, registering from 0% to 2% of the households that are supplied by water trucks and from 0% to 0.25% for rainwater storage. Considerable values for both types are only found in the interior of the state in the municipalities of Glória do Goitá and Vitória de Santo Antão, which are located more than 20 km from the study region. However, the same cannot be said about garbage disposal (Fig. S4). Recife and Olinda have low values of 0% to 3.5% for disposal in vacant land or public spaces, in great contrast with the nearby municipalities such as Jaboatão dos Guararapes, Paudalho, Camaragibe, São Lourenço da Mata and Araçoiaba, being the last one with indexes from 20% to 35%.


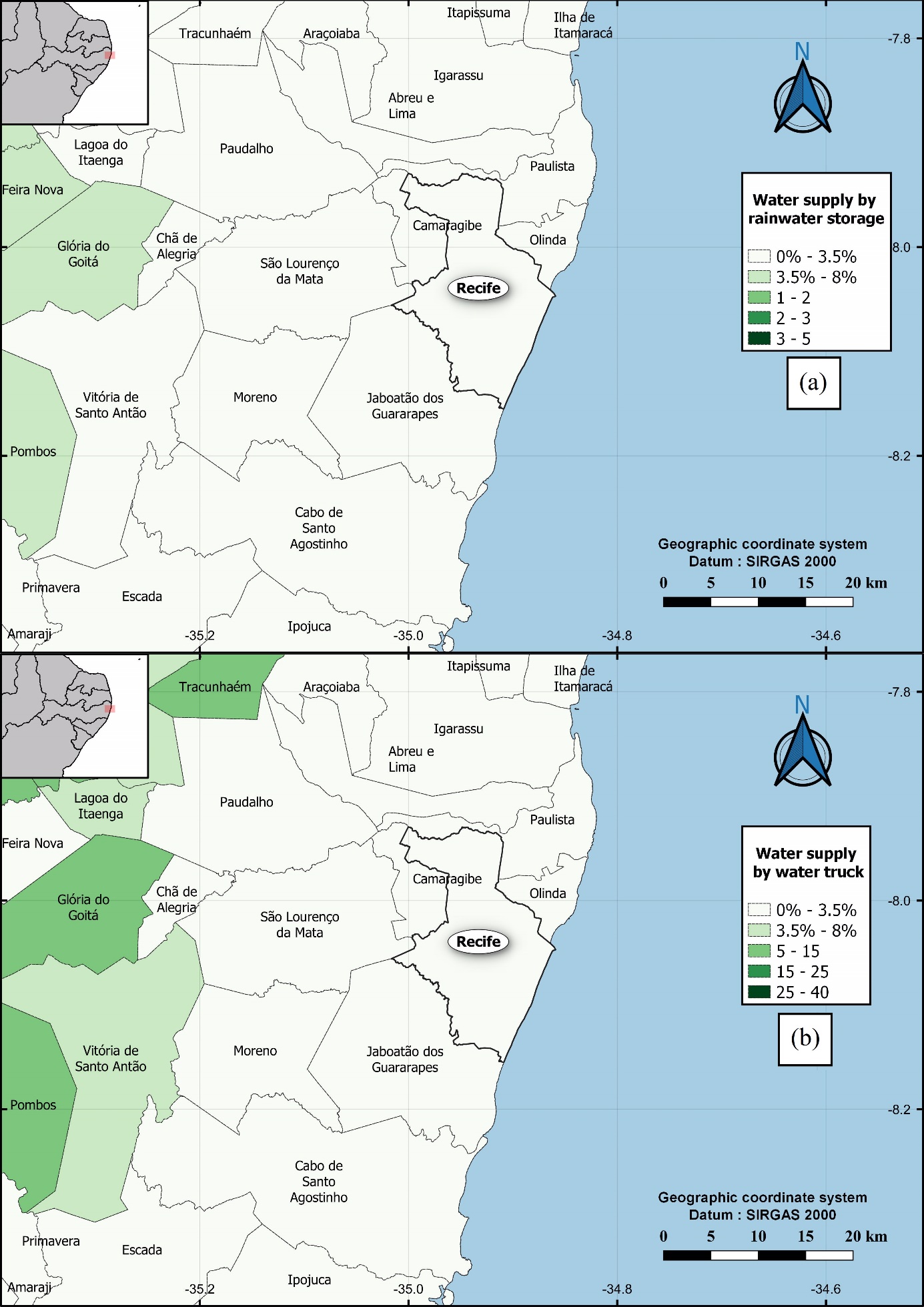


**Fig. S3** Percentage of households within the municipality in 2010 that receive water supply by **(a)** Rainwater storage **(b)** Water truck


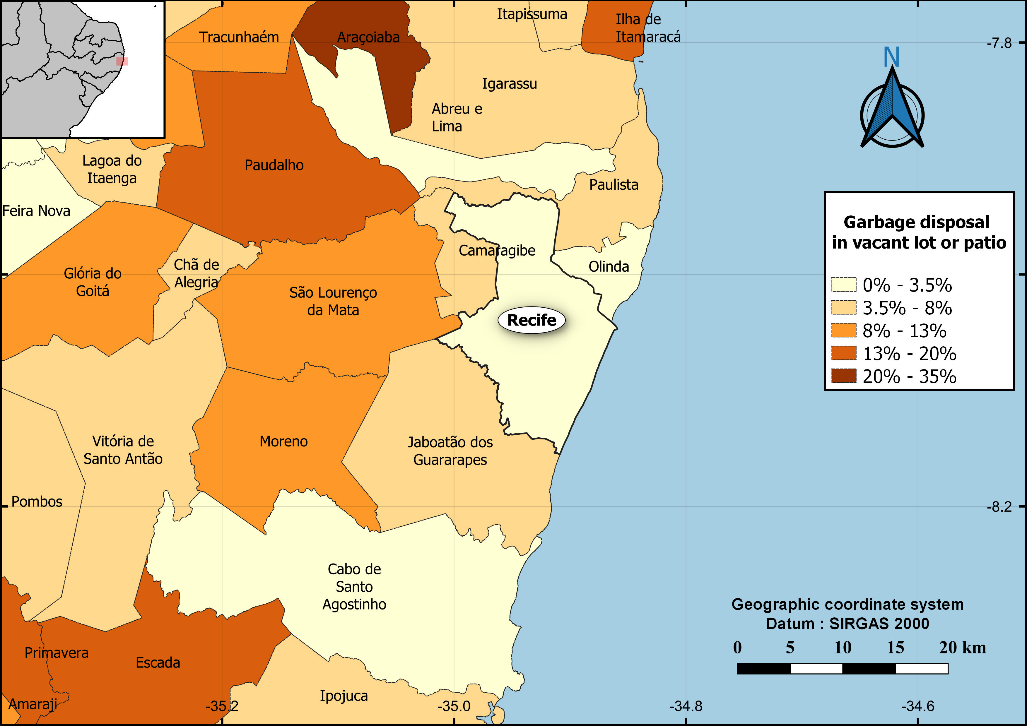


**Fig. S4** Percentage of households within the municipality in 2010 that dispose of waste in vacant land or public spaces

Although large urban centres, such as Recife and Olinda, have better sanitary conditions, their surroundings do not have these same infrastructures, causing these municipalities to end up generating more breeding grounds for the vector of transmission, in order not only to increase cases within of the municipality itself, as well as contributing to the displacement of both the virus, through the transit of infected people to regions with better conditions, and the mosquito vector of the disease itself.

Fig. S5 shows the rate of dengue cases per 100,000 inhabitants (Fig S5a) and the population density of the municipalities around Recife (Fig. S5b). The area with the highest population density is in the municipalities of Recife, Olinda, Paulista and Camaragibe, with a gradual decrease towards the interior of the state. However, despite the higher concentration of inhabitants, these municipalities do not present rates of dengue cases proportional to the demographic density, especially in Olinda and Paulista. Locations with low population density and higher case rates indicate the lack of sanitation infrastructure and adequate garbage collection, making the creation of *Aedes aegypti* breeding sites more frequent in the municipality and putting the local and nearby population at risk. In addition, it is noteworthy that, similarly, regions with high population density and low case rates point to the presence of infrastructure and mitigation measures for the mosquito population.


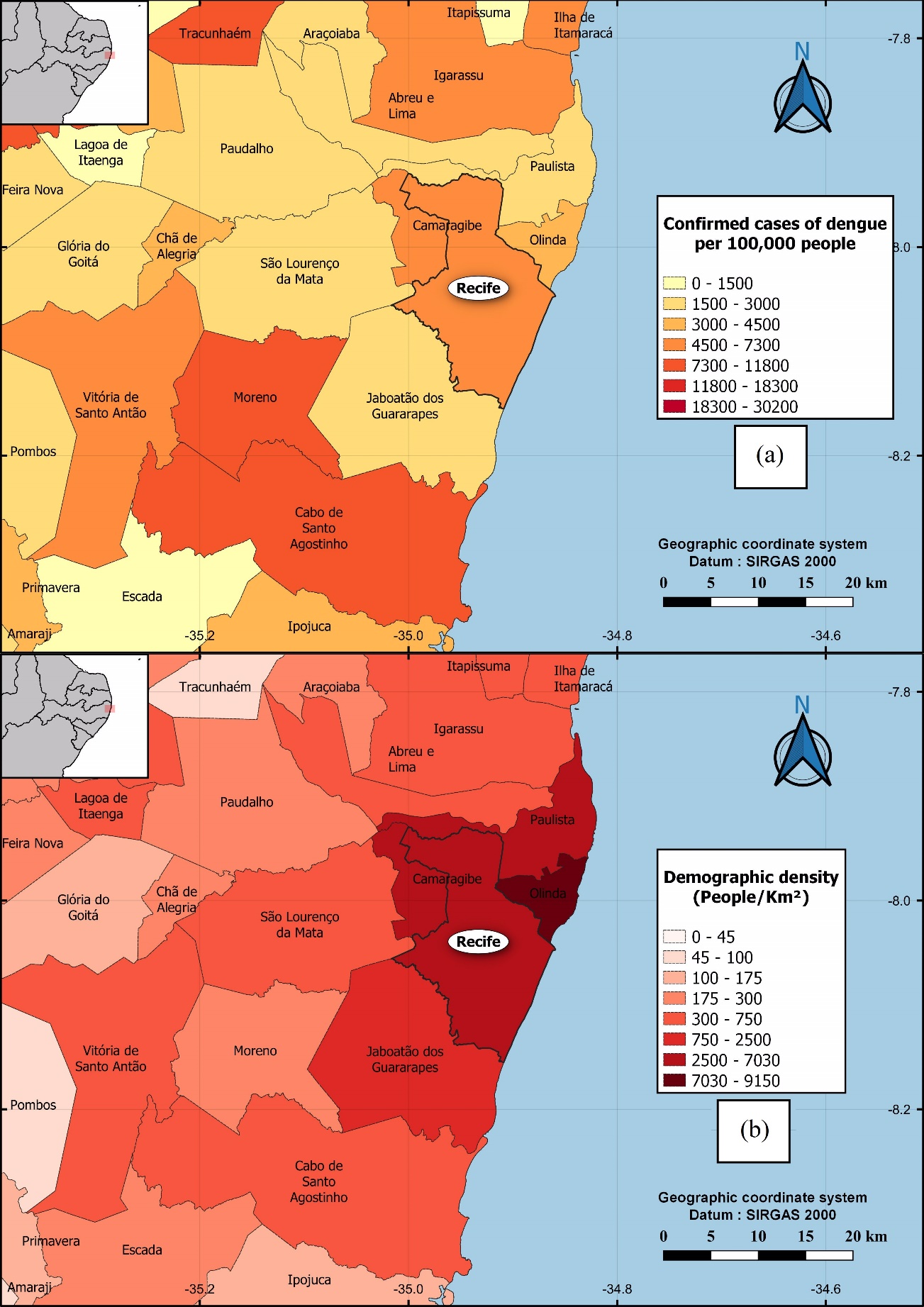


**Fig. S5** Information on the spatial distribution of the population in the study area for each municipality from the combination of data collected in the 2010 IBGE Census and DATASUS **(a)** Confirmed dengue cases accumulated in 18 years (2001 – 2019) per 100,000 people **(b)** Demographic density of municipalities

As it is a disease that depends on a transmission vector for the dissemination of cases, the high values in a given locality can only be attributed to the presence of large concentrations of vectors in the same or nearby areas, so that the emigration and immigration of people infected from great distances would not be as relevant without their presence, being more important to consider the radius of displacement of the vectors themselves.

The data referring to the type of land use (Fig. S6) show a link between the presence of artificial areas in the municipalities and case rates, a pattern expected given that *Aedes aegypti* is very adapted to urban environments. The municipality of Recife has a predominance of artificial areas and the presence of other classes such as forest vegetation, occupations in rural and forest areas and, nevertheless, continental and coastal water bodies. This heterogeneity within the municipality has the character of harming the control and application of mitigating measures in the vector populations (Teixeira et al., 1999; Mendonça et al., 2009), which perhaps explains the greater occurrence of dengue cases in this municipality. On the other hand, Olinda, which is equally dense in population, has a much lower case rate per 10,000 inhabitants and with predominantly artificial land use coverage.


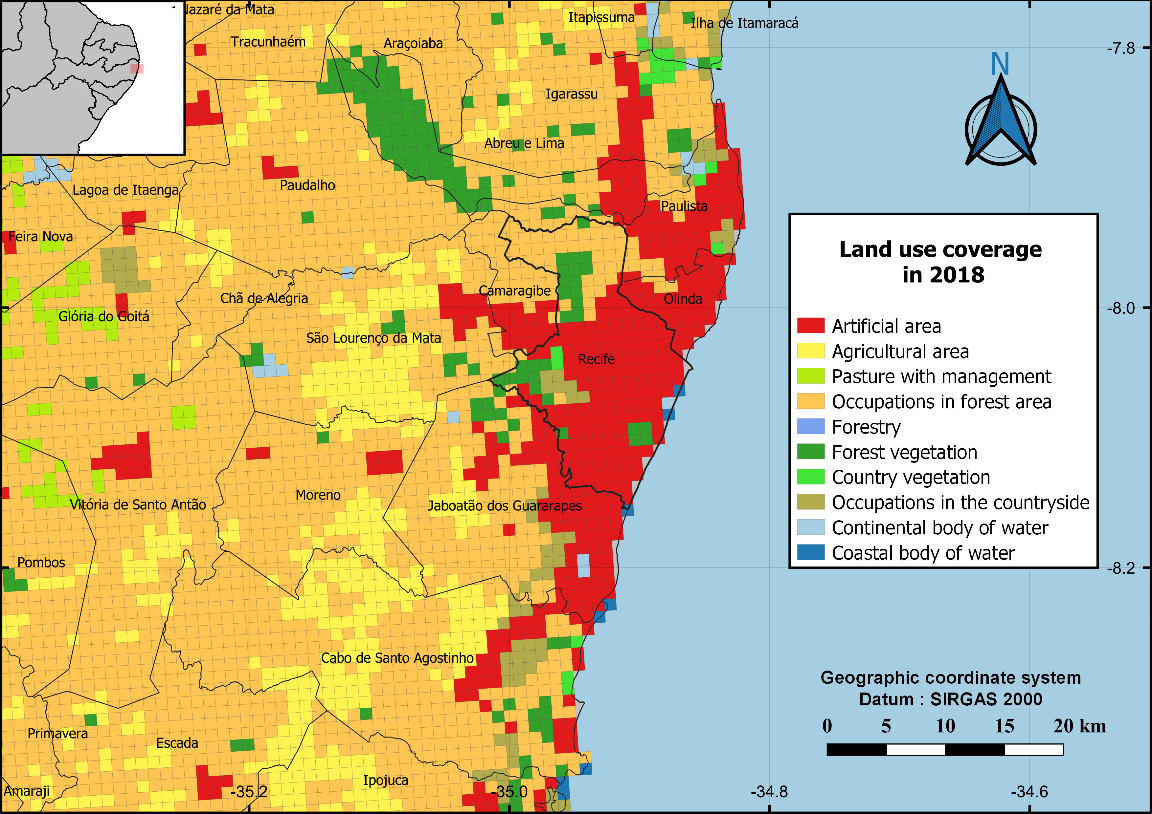


**Fig. S6** Mapping of land use through IBGE data for 2018 focused on the municipality of Recife with grids of 1 km x 1 km

From the maps showed in this study, it seems the geographic space of the city of Recife showed good sanitary conditions, both for water supply and solid waste disposal. However, the same cannot be said of neighbouring municipalities, which present favourable conditions for the formation of breeding sites for the *Aedes aegypti* mosquito. In these areas, a significant percentage of households report practicing waste disposal in vacant lots or public spaces, which, as discussed by Sobral and Sobral (2019), has a considerable impact on the increase in dengue vector populations. These unfavourable conditions can facilitate the migration of vectors to the municipality of Recife, potentially leading to an increase in cases, even when the municipality has a well-established basic sanitation infrastructure that would otherwise prevent the formation of breeding sites within its borders. Moreover, the significant heterogeneity in land cover across the municipality further complicates efforts to mitigate and control disease vectors.

**Section 4 – Precipitation Categorized**

The values considered for each quantile intervals for daily and monthly precipitation in Recife are found in tables 1S and 2S.

**Table 1S** Categorization of precipitation in Recife of the daily time series from 2001-2019 through classification by quantiles

| **Classification** | **Precipitation (mm/day)** |
| --- | --- |
| Dry day | y < 2.4 |
| Very weak | 2.4 ≤ y < 4.4 |
| Weak | 4.4 ≤ y < 8.4 |
| Moderate | 8.4 ≤ y < 19 |
| Strong | 19 ≤ y < 54 |
| Very strong | y ≥ 54 |

**Table 2S** Categorization of precipitation in Recife of the monthly time series from 2001-2019 through classification by quantiles

| **Classification** | **Precipitation (mm/month)** |
| --- | --- |
| Very dry | y ≤ 34.5 |
| Dry | 34.5 < y ≤ 84.6 |
| Normal | 84.6 < y < 210.6 |
| Rainy | 210.6 ≤ y < 354.7 |
| Very rainy | y ≥ 354.7 |

**References**

Bonita, R., Beaglehole, R., and Kjellstrom, T. (2010). EpidemiologiaBásica (Santos Editora Com. Imp. Ltda - 2.ed. - São Paulo, Santos.)

Hoffmann,  R.  (2015). Análise  de  regressão:  uma  introdução  à (ESALQ/USP) econometria  [eletronic resource]

Lima,  E. A.,  Firmino,  J. L. N.,  and Filho,  M. F. G. (2008).   A relação da previsão da precipitação pluviométrica e casos de dengue nos estados de alagoas e paraíba no nordeste do brasil.   Revista Brasileira de Meteorologia , 264–269

São Paulo. (2022). Estatísticas - Taxa por 100 mil habitantes (Portal do Governo de São Paulo. Available at: https://www.ssp.sp.gov.br/fale/estatisticas/answers.aspx?t=6. Accessed in: 23/01/2022)
